# Effects of Mechanical Deformation on the Opto-Electronic Responses, Reactivity, and Performance of Conjugated Polymers: A DFT Study

**DOI:** 10.3390/polym14071354

**Published:** 2022-03-26

**Authors:** João P. Cachaneski-Lopes, Augusto Batagin-Neto

**Affiliations:** 1 POSMAT, School of Sciences, São Paulo State University (UNESP), Bauru 17033-360, SP, Brazil; cachaneski-lopes@unesp.br; 2 Institute of Science and Engineering, São Paulo State University (UNESP), Itapeva 18409-010, SP, Brazil

**Keywords:** molecular modeling, stretching process, polymers, mechanical deformation, density functional theory

## Abstract

The development of polymers for optoelectronic applications is an important research area; however, a deeper understanding of the effects induced by mechanical deformations on their intrinsic properties is needed to expand their applicability and improve their durability. Despite the number of recent studies on the mechanochemistry of organic materials, the basic knowledge and applicability of such concepts in these materials are far from those for their inorganic counterparts. To bring light to this, here we employ molecular modeling techniques to evaluate the effects of mechanical deformations on the structural, optoelectronic, and reactivity properties of traditional semiconducting polymers, such as polyaniline (PANI), polythiophene (PT), poly (*p*-phenylene vinylene) (PPV), and polypyrrole (PPy). For this purpose, density functional theory (DFT)-based calculations were conducted for the distinct systems at varied stretching levels in order to identify the influence of structural deformations on the electronic structure of the systems. In general, it is noticed that the elongation process leads to an increase in electronic gaps, hypsochromic effects in the optical absorption spectrum, and small changes in local reactivities. Such changes can influence the performance of polymer-based devices, allowing us to establish significant structure deformation response relationships.

## 1. Introduction

Polymeric materials are very interesting compounds for several applications, playing significant roles in both applied and basic science. In particular, modern polymers have evolved into multifunctional systems wherein specific responses are expected from particular stimuli. In this context, once mechanical loads and deformations are practically inevitable, it is important to establish relationships between the mechanical properties and electronic responses of these compounds [1,2,3,4].

In fact, nowadays, mechano-responsive polymers are quite attractive for a number of applications. In these materials, mechanical forces can be used to transfer energy to chemical bonds and drive chemical reactions [5,6]. Although mechanochemistry has been known for years [5] and is already industrially employed [5,7,8,9], it has been marginally explored in organic materials compared to inorganic systems [10].

The use of organic materials for optoelectronic applications has achieved prominence; in this context, the improved mechanical properties of polymers have highlighted their possible application in organic-based flexible devices [11,12,13,14,15,16]. Composites based on elastomers and thermoplastics have provided high flexibility to these materials without considerable losses in conductivity [16,17,18], leading to devices with reasonable performance, low relative cost, and processing advantages [19,20].

As a matter of fact, among the different classes of organic compounds, conjugated polymers are promising materials for the development of flexible optoelectronic devices, such as organic solar cells (OSCs) and organic light-emitting diodes (OLEDs) [19,21,22]. However, additional studies are necessary to better understand the influence of mechanical deformations on their properties to identify relevant operating regimes/limits and obtain fully functional devices with broad applicability.

In fact, even after the insertion of flexible organic devices on the market, there is still no complete understanding of the influence of mechanical stresses on the intrinsic properties of the materials present in their active layers, which hinders the effective application of these devices. In general, the flexibility of these compounds is commonly associated with amorphous domains and interactions between chains, which rearrange themselves when subjected to external stresses [23,24,25,26]. On the other hand, the optoelectronic properties of these materials are commonly governed by planar sub-segments (and their interactions) [27,28,29,30,31], so that the influence of mechanical deformations on the response of a conjugated polymer involves a series of complex interactions [25].

A number of experimental data indicate the existence of a variety of effects on the performance of flexible devices under mechanical stresses. In general, successive deformation cycles lead to a reduction in the efficiency of OSCs [23,24,26], while high stability is reported for OLEDs [32,33]. However, the overall effects depend on the materials and processing methods [11,22,25,34]. 

Indeed, the influence of the stretching process on the optical properties of polymers is a well-known phenomenon [35] that has been revisited by several recent works focused on varied applications [36,37,38]. A number of reviews focused on mechanical-responsive functional devices and the causes of their deterioration have also been published [33,39]. For instance, Wang and collaborators [40] presented a survey of materials and techniques to obtain devices with equilibrated mechanical and electrical properties. A common point in these works is the search for an appropriate ratio between conductive and flexible materials, with minor discussions on the influence of the mechanical deformations on the intrinsic properties of the compounds.

In this context, molecular modeling techniques can be considered relevant tools to evaluate the variety of mechano-responsive processes, particularly to identify factors associated with the degradation of the optoelectronic properties, propose alternatives for their minimization, and guide the development of new materials with improved responses [41]. Such analyses are especially interesting for polymeric devices due to their high degree of structural flexibility and the strong relationship between conformational and optoelectronic properties [25,34,41,42]. 

Despite this, there is a scarcity of theoretical works on the effect of mechanical deformations on the optoelectronic properties of semiconducting polymers. Our previous studies at a moderate level of theory (semiempirical + density functional theory (DFT)) indicated that mechanical elongations of MEH-PPV and P3HT polymer chains lead to deleterious effects on the optoelectronic responses of the materials, which occurs at different regimes and levels [41]. However, the limitations imposed by the use of semiempirical approximations (for geometry optimization), and the observation of strong steric effects of the side groups, deserve further study.

To deepen such analyses, here we conduct a series of DFT-based calculations to investigate the influence of mechanical deformations on the intrinsic optoelectronic properties of four widely employed conjugated polymers. The results show that the main chain stretching leads to significant effects on the frontier energy levels of the systems, increasing the electronic gaps and leading to hypsochromic effects in the optical spectra. Such effects can influence the optoelectronic performance of polymer-based devices, mainly in systems with high molecular interactions and entanglements by polymer chains [16,22,34,43,44]. To exemplify such consequences of polymer main chain deformations, we analyze some important parameters of organic solar cells (OSCs); in particular, polymer chain deformations of around 7% and 15% can lead to non-functional PT- and PPV-based OSCs, respectively.

## 2. Materials and Methods

**Figure 1:**
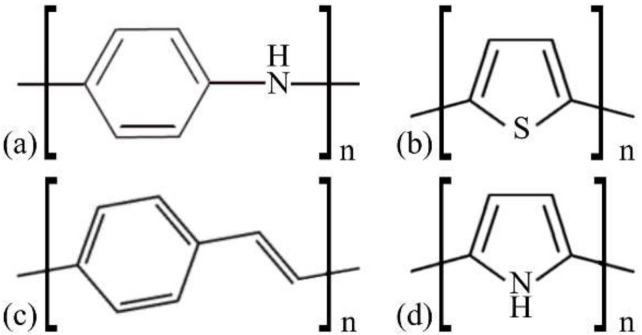
Basic structures of the polymeric materials. (**a**) polyaniline (PANI), (**b**) polythiophene (PT), (**c**) poly (*p*-phenylene vinylene) (PPV), and (**d**) polypyrrole (PPy).

Figure 1 illustrates the basic structure of the compounds evaluated in this report.

The above presented polymers were chosen according to their high potential for applications in varied devices [45]. PANI is used in electro-rheological fluids, sensors, supercapacitors and rechargeable batteries [46,47,48,49,50]. PT is widely used in polymer-based OLEDs (PLEDs), OSCs, and chemical sensors due to its conductive, luminescence, and electrochromic properties, as well as its high versatility of synthesis [51,52,53]. PPV derivatives are also commonly used in PLEDs and OSCs [54,55,56]. PPy has diverse applications due to its low relative cost, electronic properties, ease of processing, and versatility of synthesis, being widely used in the active layer of gas sensors [57,58,59,60].

Planarized oligomeric systems with average sizes around the effective conjugation lengths of these materials have been considered as representative models of the polymers. Due to the strong influence of the effective conjugation length on the optoelectronic properties of these systems [28,61], representative oligomeric systems were considered in this study. In particular, studies conducted by our group and collaborators have shown that structures with 10–15 repeating units can reproduce the essential electronic and optical properties of these polymers [47,58,62,63,64,65,66,67]; for this reason, oligomers with 15 units were used.

Preliminary stretching studies conducted for amorphous structures show a convergence of folded to planarized structures after elongation (see Supplementary Materials—in particular, Figure S1 compared to elongated structures in Figure S2), with relatively small energetic costs (see Figure S3). Thus, folded polymers are supposed to converge to planarized conformations during stretching processes. This result, associated with the fact that the optoelectronic properties of conjugated polymers are commonly governed by planar subsegments [27,28,29,30], defined planar structures as the models of interest in the present work. Given the viscoelastic behavior of polymeric systems to mechanical deformations, Figure S1 can be also considered as an indicator of shrink effects.

The initial structures were designed with the aid of the Molden 5.0 computational package [68] (details regarding the amorphous structures and their planarization process can be seen in the Supplementary Materials). The geometry optimization and calculation of electronic properties were conducted in the framework of density functional theory (DFT), using the B3LYP hybrid XC functional [69,70,71,72] and 6-31G basis set on all the atoms. The choice of such an approach was made based on the well-known capability of B3LYP to describe the structural, electronic, and optical features of medium-size conjugated systems in comparison with other functionals [73], associated with preliminary calculations conducted for distinct approaches which indicated that the effects of mechanical deformations are not so sensitive to the XC functionals and basis sets employed (see Table S3 in the Supplementary Materials for additional calculations/considerations). In addition, given that we are not interested in the precise reproduction of absolute values, but rationalizing the relative changes induced by the deformations, we considered the B3LYP/6-31G approach to be a reasonable choice due to the relative cost–benefit ratio. All the calculations were conducted with the aid of the Gaussian 16 computational package [74].

**Figure 2:**
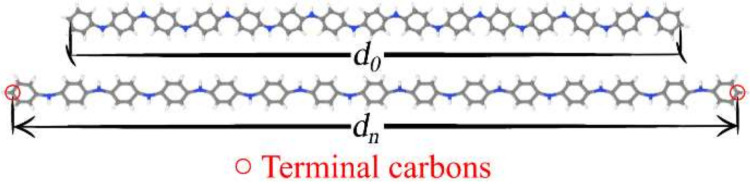
Illustration of the polymer chain deformation process (example for PANI).

The mechanical deformation processes were performed considering the fully optimized structures as starting geometries (without restrictions). The main chain stretches were then made by displacing the terminal carbons of the oligomeric structures (initially at distance *d*_0_, see Figure 2), defining new relative positions between them (*d_n_*) [75]. For instance, at Step 5, we obtained *d*_5_ = 1.05*d*_0_, i.e., an imposed elongation of 5% of the chain in relation to the (pre-optimized) relaxed structure. The mechanical deformation process imposed on the oligomers is exemplified for PANI in Figure 2. All the structures were subjected to gradual elongation until their rupture.

(1) $$d_{n}=d_{0}\left(1+\frac{n}{100}\right)$$

The resulting structure was then allowed to relax (geometry optimizations), restricting the modified distances *d_n_* (Equation (1)) (for *n* steps). The partial geometry relaxation of all the structures (restricting *d_n_* for each step) was performed via a DFT/B3LYP/6-31G approach with the aid of the Gaussian 16 computational package.

The effects of structural changes on the opto-electronic properties of the systems were evaluated considering the: (i) electronic gaps, (ii) energetic and spatial distribution of the frontier molecular orbitals (FMOs); (iii) total density of states (DOS); (iv) optical absorption spectra; (v) exciton binding energies, vi) internal reorganization energies, and (vii) local reactivities.

The electronic gaps (*E_gap_*), DOS, and FMO energies for the highest occupied (HOMO) and lowest unoccupied (LUMO) molecular orbitals were estimated from the Kohn–Sham orbitals. The theoretical optical absorption spectra were obtained via a time-dependent DFT (TD-DFT) approach using the same functional and basis set employed in the geometry optimizations. Ten transitions were evaluated, considering only single excitations. The exciton binding energies (*E_X_*) (Equation (2)) were estimated from the difference between the fundamental gaps (from KS-FMOs) and the vertical transition energies (*E_vert_*) resulting from TD-DFT calculations [41,76,77].

(2) $$E_{x}=\left(E_{LUMO}-E_{HOMO}\right)-E_{vert}$$

The internal reorganization energies for electrons (*λ_e_*) and holes (*λ_h_*) were evaluated via Equations (3) and (4). These parameters indicate the energy penalties due to the structural relaxation of the molecules (internal contribution *λ^int^*) and polarization effects on the surrounding medium (external contribution *λ^ext^*) associated with the charge transfer processes between planar (conjugated) subsegments of the materials [78]. In fact, it is well known that, despite having some effect in charge transport, *λ^ext^* is very small in relation to *λ^int^*, being often neglected [65,79]. Thus, in this work the reorganization energy was approximated by *λ* ≈ *λ^int^*, i.e.,

(3) $$\lambda_{h}=\left[E_{T}\left(\nu_{N-1},N\right)-E_{T}\left(\nu_{N},N\right)]-[E_{T}\left(\nu_{N},N-1\right)-E_{T}\left(\nu_{N-1},N-1\right)\right]$$

(4) $$\lambda_{e}=\left[E_{T}\left(\nu_{N+1},N\right)-E_{T}\left(\nu_{N},N\right)]-[E_{T}\left(\nu_{N},N+1\right)-E_{T}\left(\nu_{N+1},N+1\right)\right]$$

For a system *M* with *N* electrons, *E_T_*(*ν_N + k_*, *N + j*) represents the total energy obtained from single-point calculations for the species *M^-j^* (i.e., *M* with *N + j* electrons) with structure (defined by *ν_N + k_*) previously obtained from the optimization of the *M^-k^* species (i.e., *M* with *N + k* electrons) [41]. In general, low values of *λ_e_* (*λ_h_*) indicate that the transport of electrons (holes) is facilitated in the materials.

The local reactivities of the compounds were evaluated via condensed-to-atoms Fukui index (CAFI) values [80,81,82,83] and molecular electrostatic potential (MEP) maps. CAFI values were estimated from the finite difference of the atomic populations considering the Hirshfeld partition charge [84,85,86], and MEP maps were generated considering the CHelp partition charge scheme [87]. The graphical representations were created with the aid of the Jmol [88] and Gabedit [89] computational packages.

The mechanical parameters were estimated for all the levels of deformations considering the derivative of the change in the total energy (Δ*Etotal* = *Etotal*^(*n*)^ − *Etotal*^(0)^) in relation to the linear deformations (*x* = *d_n_* − *d*_0_). Thus, the amplitude of the forces (|*F*|) required for each level of deformation was estimated by |*F*| = *d*Δ*E_total_/dx*. The elastic constants (*k*) were estimated via linear fittings, by considering |*F*| = *k.x* [90] (see the Supplementary Materials for details).

## 3. Results and Discussion

### 3.1. Structural Changes

Table 1 summarizes the structural data of the systems before and after the deformations. *d_0_* and *d_n_* represent the distances between the terminal carbons of the chains before the deformations (equilibrium position) and at the point of imminent rupture. Δ*d_max_* represents the maximum percentage change before the oligomer break. PT and PPV presented slightly distorted (twisted or curved) initial structures (see Supplementary Materials).

**Figure 3:**
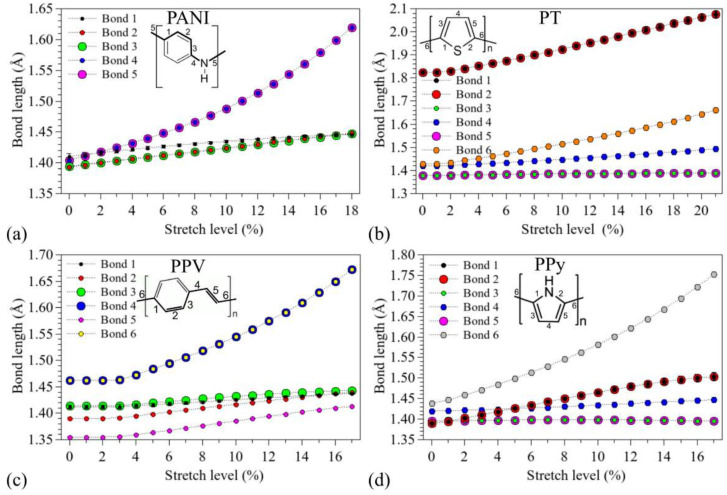
Changes in the oligomer bond lengths during the stretching processes: (**a**) PANI, (**b**) PT, (**c**) PPV, and (**d**) PPy.

**Figure 4:**
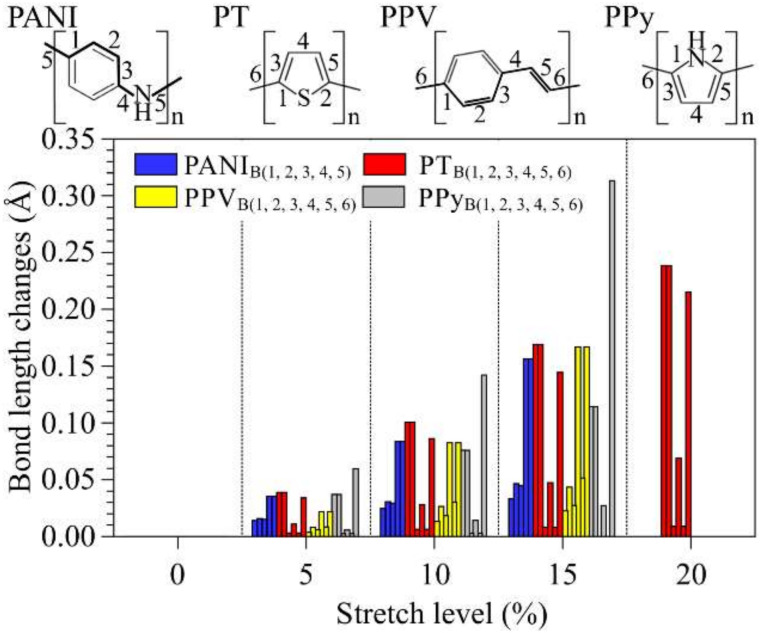
Summary of the changes in polymer bond lengths at distinct stretch levels.

Figure 3 illustrates how the chemical bonds of the systems (numbered in the insets) changed after successive stretches (the bond lengths at *d_0_* are compatible with those presented in the literature [91,92,93,94], see the Supplementary Materials). The relative variations are summarized in Figure 4.

The following order of bond lengths (BL) was noticed for non-modified PANI: BL_1_ > BL_4_ ~ BL_5_ > BL_2_ ~ BL_3_. It is possible to note an exponential increase in BL_4_ and BL_5_ (with total changes of up to 0.22 Å), while the other connections show variations of up to 0.05 Å. We note that BL_1_ tends to approach BL_2_ and BL_3_ during stretching, leading to uniform bonds in rings after 17%. For PT, minor increases are noted on BL_3_ and BL_5_. Although the oligomer disruption occurs at Bond 6, the highest stretches were noticed for BL_1_ and BL_2_ (up to 0.24 Å). Very small changes were observed for BL_4_ (<0.05 Å). PPV presents significant changes only after the 4% stretch, after the planarization of the structures (see Figure S2c). Major changes were noticed for BL_4_ and BL_6_ (~0.21 Å). As noted in the case of PANI, for PPV, the bonds on the rings are uniform at high deformation levels. Finally, for PPy, we noticed an exponential increase in BL_6_ (~0.31 Å), with a tendency of ring opening (increase in BL_1_ and BL_2_) and minor variations in BL_3_, BL_4_, and BL_5_.

Similar results were reported by Rodao et al. [41] for P3HT and MEH-PPV using semi-empirical methods for geometry optimization. In particular, for MEH-PPV (P3HT), an exponential increase was observed for single C–C bonds (C-S) with small variations in the benzene rings (C=C).

The above presented results allow us to underline the dominant degradation routes for each system. For instance, it is noted that the major stretches of PANI occur through nitrogen–carbon bonds (BL_4_ and BL_5_), while PPV presents significant stretches on the carbon bonds around the vinylene units; we note that the mechanical deformation of these systems does not significantly alter the C–C bonds inside the rings (i.e., BL_i_ < 1.45 Å). A tendency to ring opening was noticed for compounds containing five-membered heteroaromatic rings (PT and PPy); this is more evident for PT, where BL_1_, BL_2_, and BL_6_ present very similar deformation rates. Although the PPy rings were not severely modified, significant changes are noted in relation to typical C–N bond lengths [95] (i.e., BL_1_ and BL_2_ > 1.309 Å).

Table 2 summarizes the relative forces required to deform the systems (*F* = |*F*|), as well as the elastic constants (*k*) estimated at each deformation level for distinct systems.

We note that the forces required to break the polymer chain are compatible with those reported for covalent bonds [75,96,97,98,99]; in particular, the following order was noticed: PPy < PT < PANI < PPV, with *Fmax* ranging from 6.84 to 8.76 nN (see Supplementary Materials for details).

### 3.2. Changes in Opto-Electronic Properties

**Figure 5:**
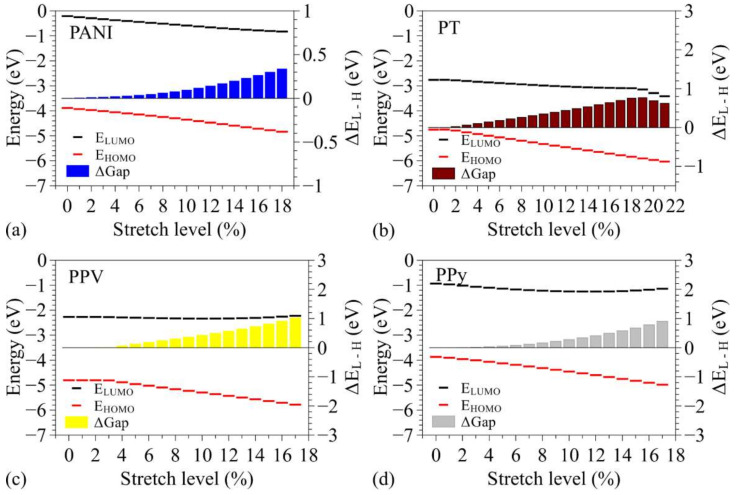
Evolution of the FMO energy levels with stretching of the oligomers: (**a**) PANI, (**b**) PT, (**c**) PPV, and (**d**) PPy.

Figure 5 illustrates how the energies of the FMO levels (*E_HOMO_* and *E_LUMO_*) vary as a function of the stretching levels. It also presents the changes induced in the electronic gaps (*E_L-H_* = *E_LUMO_* − *E_HOMO_*) in relation to those for the unstretched system (Δ*E_L-H_*). The numerical values for non-stretched structures are presented in the Supplementary Materials (Table S2).

In general, we noticed a reduction in the FMO energies with stretching for all the systems. The changes are more pronounced in the *E_HOMO_* values, leading to an increase in the electronic gaps. The effects noticed for PT are partially compatible with the results reported for P3HT by Roldao et al. [41], mainly in relation to *E_LUMO_*. We considered that the anomalous behavior of *E_HOMO_* presented in ref [41] is associated with the steric effects of the side groups; these have a profound influence on the dihedral angles of the main chains, which is not present in our calculations. This interpretation is reinforced by the absence of transitions around 14% for PPV, which were reported for MEH-PPV in ref [41] and associated with the interaction between adjacent ramifications. For PT, a sharp drop was noted in *E_LUMO_* after 20% stretching, which is associated with the saturation of the stretching process, whereafter the system behaves as a set of non-passivated thiophene units (see Figure S7). 

For PANI, we noted that FMO levels vary linearly with the deformation level of the chains, presenting very similar rates for *E_HOMO_* and *E_LUMO_* changes, leading to small variation of the gaps (lower than 0.4 eV). Given the initial structural distortion of fully relaxed PT (see Figure S2b), their FMO levels remained unchanged until 2% stretching and then presented a decrease, leading to a gradual increase in the electronic gaps (of up to ~0.77 eV); a change in the behavior of the LUMO was noted after 19% stretch with a more pronounced decrease rate. Due to the initial distortion of fully relaxed structures of PPV, no variations could be observed for this compound until 4% stretching (see Figure S2c). After this, both the FMO levels were reduced with more expressive (and linear) effects on the HOMO, leading to increased electronic gaps (of up to 1 eV); after 11% stretching, we noted a change in the behavior of the LUMO, showing a tiny increase with stretching. Finally, for PPy, we initially noted a reduction of FMOs at similar rates; the behavior of the LUMO changed after 5% (lower decreasing rate) and 11% (when it started to increase).

**Figure 6:**
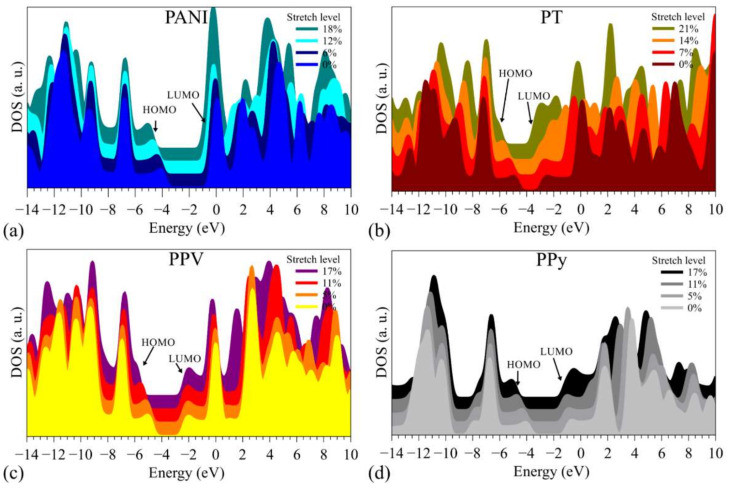
DOS for (**a**) PANI, (**b**) PT, (**c**) PPV, and (**d**) PPy at distinct stretch levels.

Figure 6 shows the changes induced by the stretching on the density of states (from KS eigenvalues) around the FMOs.

In general, significant variations were noted far away from the frontier levels, with subtle changes around the HOMO and LUMO (mainly in the LUMO) (see the Supplementary Materials for more details).

**Figure 7:**
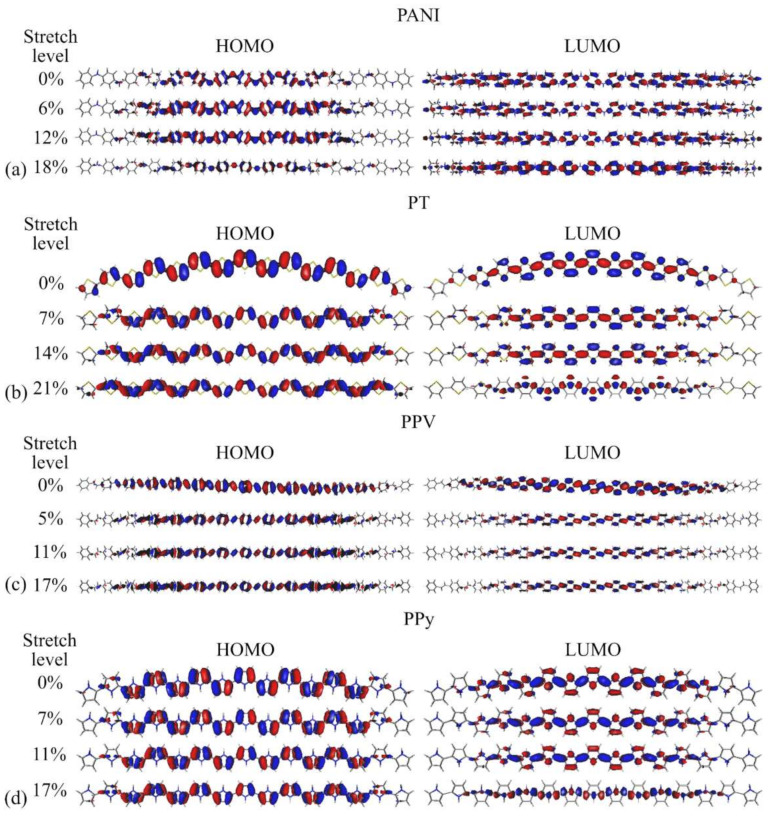
Effects of mechanical stretching on the spatial distribution of the KS-FMOs of (**a**) PANI, (**b**) PT, (**c**) PPV, and (**d**) PPy.

Figure 7 presents the spatial distribution of the HOMO and LUMO KS orbitals for some representative stretching levels.

For all the systems, we noted that HOMOs are transversely aligned to the polymer main axis, while the LUMO lobes are parallel to them. The stretching process does not have a strong influence on the spatial distribution of the FMOs, leading to significant changes (localization of the wavefunctions) only when rupture is imminent, mainly on the LUMO of PT, PPV, and PPy and on the HOMO of PANI, degrading their optoelectronic properties. An opposite effect was noticed for the HOMO (LUMO) of PT (PANI), for which higher delocalization was noticed in very distorted structures, indicating improved hole (electron) transport in the stretched systems. PPV is more sensitive to small deformations in relation to the other structures; however, it does not show intense degradation at high levels of deformation. A similar behavior of the LUMO was noticed in very distorted structures of PT and PPy (Figure 7b,d). In general, the results suggest that deterioration of the opto-electronic properties occurs for most of these systems after 11% stretching. 

**Figure 8:**
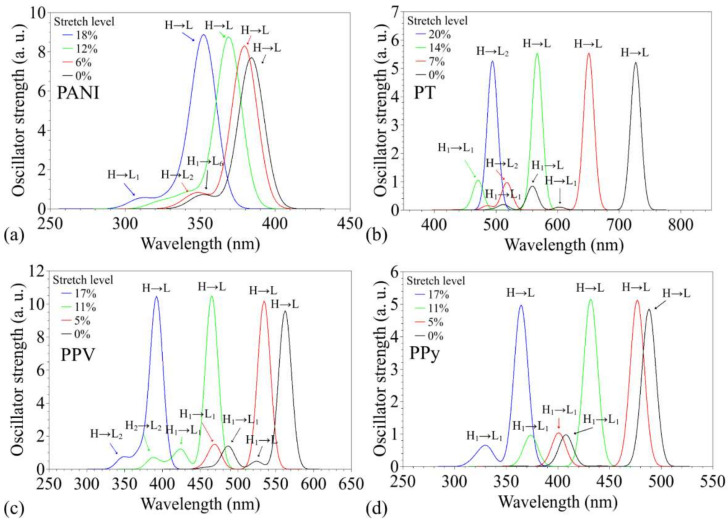
Summary of the changes on the optical absorption spectra of the polymers during the stretching process: (**a**) PANI, (**b**) PT, (**c**) PPV, and (**d**) PPy.

Figure 8 demonstrates the behavior of the optical absorption spectra of the compounds throughout the deformation process.

Hypsochromic effects were noticed for all the structures, and these are compatible with the relative increase in the electronic gaps presented in Figure 5. Stronger effects were noticed for PT (Δ*λ_max_* ~233 nm) and PPV (Δ*λ_max_* ~170 nm), with blueshifts that are approximately linear with regard to the deformation level; less intense (but non-linear) blueshifts were noticed for PPy (Δ*λ_max_* ~124 nm) and PANI (Δ*λ_max_* ~32nm) (see Figure S6). For all the cases, the main peaks are governed by HOMO–LUMO transitions along the deformation processes, except for PT at the point of imminent rupture (with a significant HOMO–LUMO + 2 contribution). The blueshift of the main peaks is followed by slight increases in the relative amplitudes, which was continuous for PANI until rupture. The small effect on the oscillator forces is compatible with the small effects noticed on the spatial distribution of the FMOs (and then on their superpositions), as presented in Figure 7. In particular, it is possible to note the appearance of secondary absorption peaks at shorter wavelengths during the stretches (see Tables S4–S7 for details); these are associated with transitions involving a variety of levels around the FMOs (HOMO-n and LUMO+m) and are compatible with the intensification of the DOS around the frontier levels.

In fact, significant blueshift in the absorption spectra of polymer-based flexible devices has already been reported in the literature [35,95], being primarily associated with the disruption of aggregates in the active layer of the devices. Our results suggest that a similar effect can take place due to the reduced electronic coupling between adjacent units.

**Figure 9:**
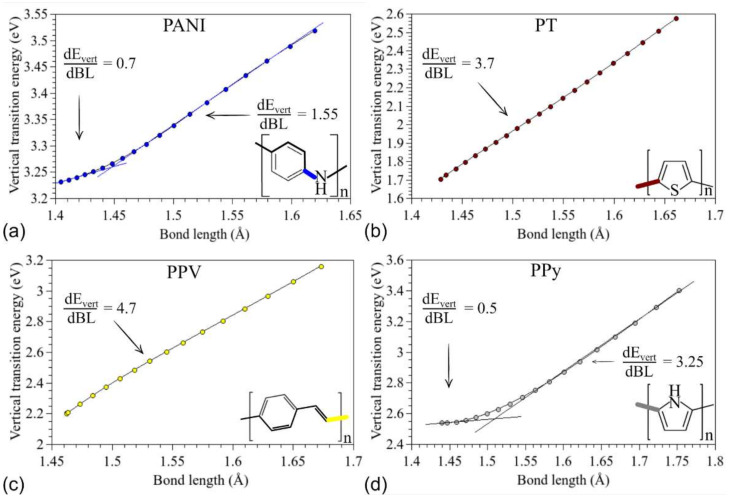
Relationships between the vertical transition energies and the most affected connections in the stretching process: (**a**) PANI, (**b**) PT, (**c**) PPV, and (**d**) PPy.

To better understand the effect of the main chain deformations on the optical properties of the compounds, Figure 9 presents the relationship between the chemical bond lengths (BL) that are broken during the rupture (highlighted in the insets) and *E_vert_* values.

In general, there is a linear dependence between the vertical transition energies and the considered bond lengths. Such a dependence is more evident for PT and PPV. In the case of PANI and PPy, a transition is noted at ~1.45 Å and ~1.5 Å, respectively, from which point they start to present a linear behavior similar to that of the other systems. This effect, associated with those presented in Figure 8, suggests that the optical absorption of the systems can be modulated via mechanical deformations. In particular, deformations smaller than 10% can lead to changes of around −15 nm (PANI), −112 nm (PT), −97 nm (PPV), and −56 nm (PPy) in the position of the absorption mean peak, without significant changes in the optical performance.

**Figure 10:**
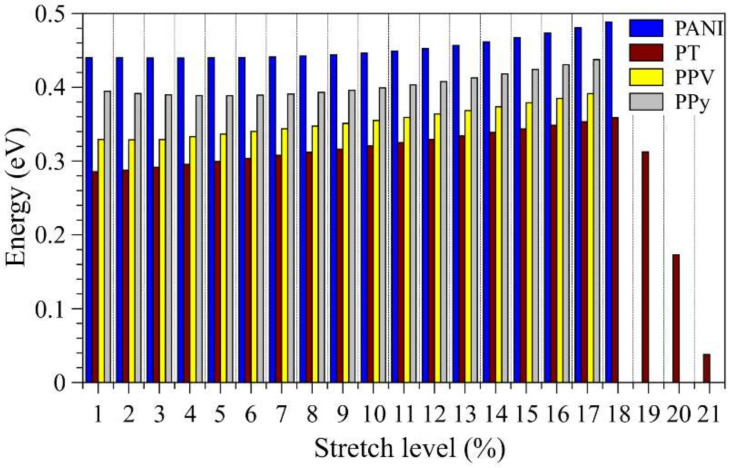
Evolution of the exciton binding energies during the stretching processes.

Figure 10 presents the changes induced in the exciton binding energies due to stretching of the polymer main chains.

The *E_X_* values of the non-modified systems are compatible with those expected for organic polymers (i.e., around 0.3 eV [100,101,102]); in particular, the order *E_X_*(PT) < *E_X_*(PPV) < *E_X_*(PPy) < *E_X_*(PANI) was noticed, which evidences the applicability of PT and PPV in OSCs. For all the systems, we observed a gradual increase in the exciton binding energies, even with distinct rates, until 17%. In addition, for PT, we noticed a rapid decrease from 18% onwards, which is in line with the changes in the LUMO levels (Figure 5).

**Figure 11:**
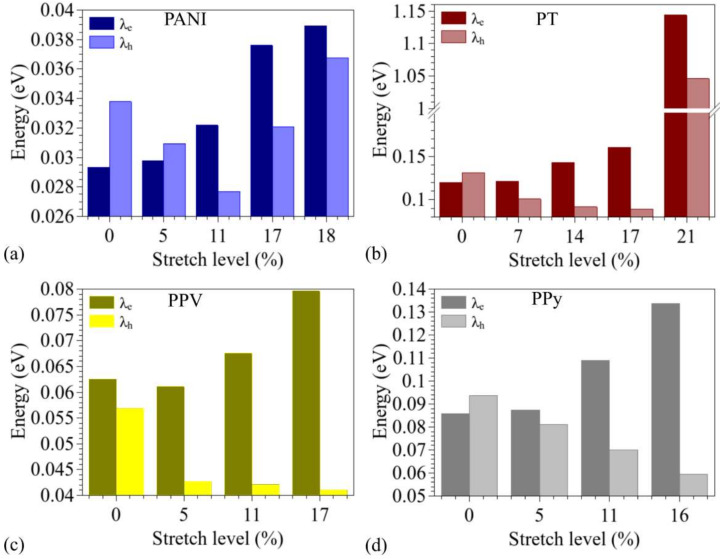
Evolution of the internal reorganization energies during the stretching process: (**a**) PANI, (**b**) PT, (**c**) PPV, and (**d**) PPy.

Figure 11 presents the changes induced in the internal reorganization energies during the stretching processes for the distinct polymers. For most systems, it shows the results obtained for up to 16% stretch, due to convergence problems in very distorted cationic structures.

The non-modified structures presented small reorganization energies (from 1 to 5 times the thermal energy at room temperature, kT_300_). Very small energies were noticed for PANI, followed by PPV, PPy, and PT. For most systems, the *λ_e_* values are slightly lower than *λ_h_*, except for PPV. During the stretching process, we noticed a gradual reduction in *λ_h_* values for all the systems, which was continuous for PPV and PPy until rupture. PANI and PT presented minimum values of *λ_h_* at 11 and 17%, respectively, with a significant increase thereafter. On the other hand, for all the systems, we noticed a gradual increase in the *λ_e_* values throughout the stretching process. These results indicate that mechanical deformations can improve (reduce) the hole (electron) transport in these materials. 

**Figure 12:**
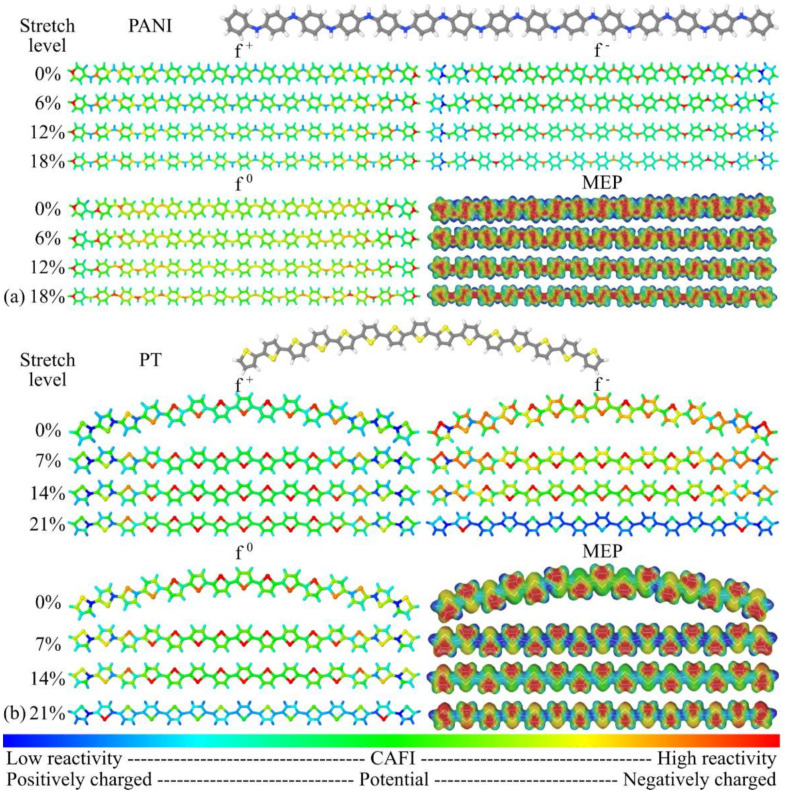
CAFI and MEP maps at distinct stretch levels: (**a**) PANI and (**b**) PT.

**Figure 13:**
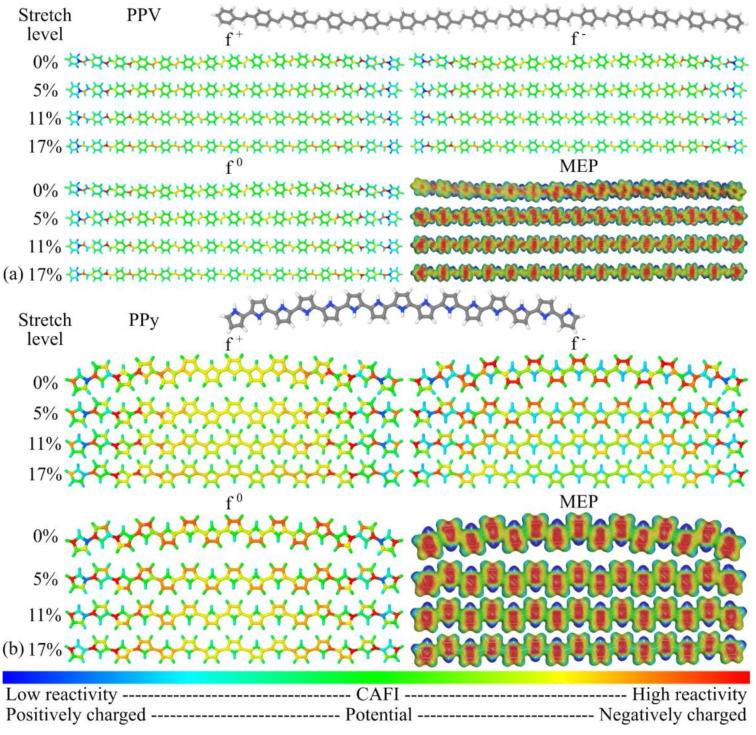
CAFI and MEP maps at distinct stretch levels: (**a**) PPV and (**b**) PPy.

CAFI and MEP analyses were conducted to evaluate the influence of mechanical stretching on the local chemical reactivity of the systems. CAFI indicates which molecular sites are prone to receive/donate electrons from/to the environment, defining which regions are susceptible to undergoing chemical reactions towards nucleophiles (*f*^+^), electrophiles (*f*^−^), and free radicals (*f*^0^). MEPs, on the other hand, indicate the charge concentration on oligomer structures, which plays a relevant role in electrostatic interactions and drives effective molecular collisions for chemical reactions. The CAFI and MEP analyses during the stretching process allow us to evaluate how “soft–soft” (i.e., associated with deformations in the frontier orbitals) and “hard–hard” (guided by electrostatic effects) interactions [103] could change due to the continuous deformation of the systems. Figure 12 and Figure 13 illustrate the CAFI and MEP colored maps for PANI, PT, PPV, and PPy. Red and blue regions define reactive (negatively charged) and non-reactive (positively charged) sites on the molecules via CAFI (MEP) analysis, respectively.

The influence of mechanical stretching on the stability of organic devices is generally discussed in terms of macroscopic/morphological features, such as delamination of the layers (active and transporting layers, as well as electrodes), the formation of punctures and cracks, strong lateral strains, limitations imposed by brittle electrodes, etc. [104]. However, a number of nanoscopic mechanisms for stress-induced polymer degradation have also been proposed [105]. In particular, the exponential behavior of the strongly stretched bonds (Figure 3) is compatible with Zhurkov-like evolution of the photochemical degradation rates of stretched polymers. 

From the analysis of Figure 12 and Figure 13, the reactivity of PANI (in relation to nucleophiles) is mainly located at terminal regions, which can be associated with the enhanced electropolymerization properties of this polymer [106]. High reactivity is observed on the nitrogen atoms, in relation to electrophiles, which is associated with the doping process of leucoemeraldine [107]. PT presents high reactivity on the sulphur atoms for both nucleophiles and electrophiles, which indicates the plausibility of *p*- and *n*-doping via this site; this is compatible with other works [108,109]. PPV presents high reactivity on the vinyl groups for all the reactions; this is not sensitive to stretching and can explain the well-known degradation routes of the polymer chains via varied mechanisms [62,110,111,112]. Finally, PPy shows high reactivity on positions 3–4 (towards electrophiles) and on position 2 (towards nucleophiles), which are compatible with the charge transfer mechanisms/doping processes proposed in the literature [108,113,114].

In general, it was noticed that *f ^+^* is very insensitive to mechanical stretching, indicating that chemical reactions towards nucleophilic agents are not changed due to the deformation of the polymer chains. On the other hand, a slight decrease in the reactivity for *f*^−^ and *f*^0^ at central regions of the chains was noticeable during the stretching process, which influences the chemical reactivity towards electrophiles and free radicals. The most significant variation in the MEPs during the stretching process was the decrease in the electronic density between the monomeric units of the main chains.

### 3.3. Considerations Regarding the Effect of Stretching on the Performance of the Compounds in Devices and Identification of Operational Regimes

Several factors can influence the efficiency of organic devices, mainly in relation to charge transport, the formation of interfaces, solubility issues, oxidation stabilities, morphologies, processing, and material synthesis [41,115,116]. However, on a nanoscopic scale, some molecular parameters, particularly the FMO level alignment, are intrinsically associated with the performance of organic devices [116,117,118], governing a number of relevant mechanisms.

In recent years, a number of studies have been conducted to evaluate the effects of stretching on polymer-based OSCs [43,44,119,120]. Polymeric systems present a viscoelastic response to mechanical deformations which depends on the polymer structure, loading rates, and working temperatures. In particular, in these systems, there is competition between chain deformation and contraction due to the stress and entropic responses [34]. As a result, the dynamics of polymer-based thin films are generally governed by the relative slippage of adjacent chains. However, effective chain entanglements can also occur in the films (mainly those based on longer polymers), resulting in large plastic deformation prior to fractures, which are associated with polymer deformation, chain pullout, or even polymer scission [44,120]. In this context, to exemplify the effects of such mechanical deformations on the performance of OSCs, here we present a simple prediction of some electronic descriptors of interest.

**Figure 14:**
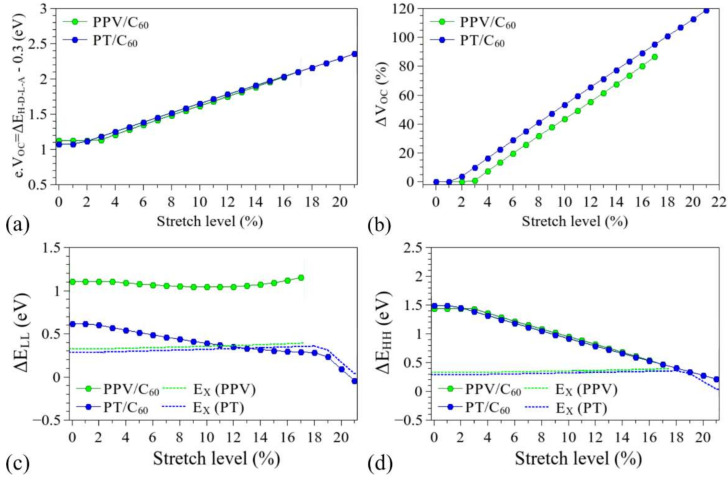
Evolution of the parameters (**a**) *V_OC_*, (**b**) Δ*V_OC_*, (**c**) Δ*E_LL_*, and (**d**) Δ*E_HH_* during the stretching processes for C_60_-based devices.

**Figure 15:**
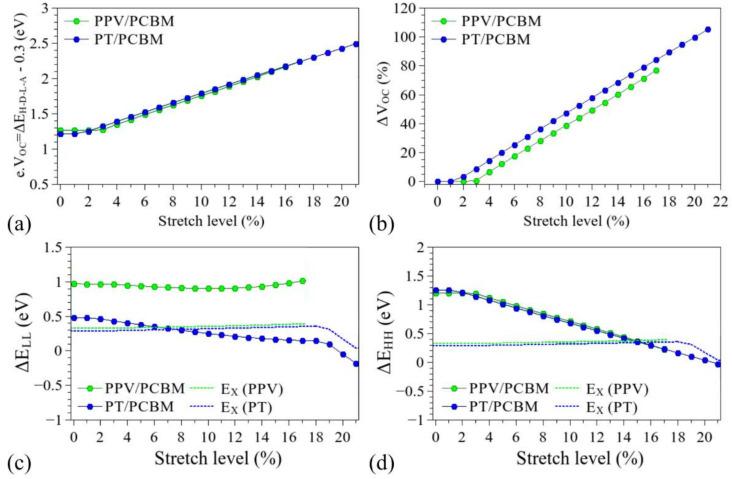
Evolution of the parameters (**a**) *V_OC_*, (**b**) Δ*V_OC_*, (**c**) Δ*E_LL_*, and (**d**) Δ*E_HH_* during the stretching processes for PCBM-based devices.

Figure 14 and Figure 15 show the influence of the structural deformation of PPV and PT (commonly used in BHJ-OSCs) on *V_OC_*, Δ*E_HH_*, and Δ*E_LL_*, considering the frontier levels of typical electron acceptors, C_60_ and PCBM, computed at the same level of theory [121]. The dotted line indicates the limits for which the parameters Δ*E_HH_* and Δ*E_LL_* are lower than the typical exciton binding energies of the polymeric donors. Details on the changes induced in the relative alignments between the FMOs are presented in the Supplementary Materials (Figure S11).

As stated before, the mechanical deformation has a strong influence on the FMOs of the structures. In particular, considering that the electron donation process is mainly governed by the relative positions of the donor/acceptor LUMO levels (LUMO_D_ and LUMO_A_), the decrease induced in the LUMO_D_ (see Figure 5 and S11) tends to reduce the *∆E_LL_* values, hindering the dissociation of the excitons in the systems [21,41]. In addition, the reduction of the donor HOMO (HOMO_D_) tends to reduce the *∆E_HH_* values, facilitating the reassociation of electron–hole pairs. Such effects prevent the effective generation of free charge carriers in the devices, leading to loss of functionality for *∆E_LL_* < *E_X_* and *∆E_HH_* < *E_X_*. In particular, it is noted that these limits are reached for deformations of around 13% and 7% for the PT/C_60_ and PT/PCBM systems, respectively. PPV/C_60_-based systems are supposed to keep their functionality throughout the deformation process, while PPV/PCBM systems present operational conditions for up to 15% stretching.

The structural changes can also lead to an increase in Δ*E_H-D-L-A_* (i.e., LUMO_A_-HOMO_D_) which can lead to higher *V_OC_* with stretching (of up to 100%). Such an interesting feature, however, is limited by the previously discussed difficulty of charge carrier generation (*∆E_LL_* < *E_X_* and *∆E_HH_* < *E_X_*).

The increase in *V_OC_* is compatible with the results reported for P3DDT/PCBM BHJ-OSCs. Savagatrup et al. [23] indicated improved performance (particularly with higher *V_OC_*) of such PT derivative-based devices when they are subjected to stretching. Interestingly, an opposite effect was noticed for P3HT/PCBM, which is associated with the presence of morphological changes in the films (formation of visible cracks). In fact, such macroscopic defects are supposed to govern the performance of this system to the detriment of local deformation of the chains, degrading the electrical response of the devices. On the other hand, the presence of long side chains in P3DDT derivatives (dodecyl groups) improves the mechanical properties of the films, so that the effect of chain deformations (also associated with chain entanglements) can play a relevant role in P3DDT/PCBM devices. Savagatrup et al. [23] reported an improvement of 14% in the *V_OC_* after stretching of 10% of such devices; according to our results, this is linked to an effective elongation of about 3-4% of the PT chains (see Figure S9b), for which we predict an operational BHJ-OSC (considering the *∆E_LL_*, *∆E_HH_*, and *E_X_* relations).

In general, the effects of deformation on the LUMO of the polymers can lead to reduced charge injection barriers from electrodes. In particular, the pronounced effects for the HOMOs (0.95, 1.28, 0.98, and 1.11 eV for PANI, PT, PPV, and PPy, respectively) can lead to non-equilibrium electrode/organic layer alignments in very stretched systems. The significant changes noticed on the FMOs are also supposed to have a profound effect on the performance of these materials in chemical sensors, given the relative position of the electronic gaps of the polymers and the FMO levels of the analytes [122,123].

It is worth considering that despite our studies having been conducted until the rupture of the chains, much smaller deformations are supposed to occur in real devices, mainly due to the mechanical restrictions imposed by the electrodes [124] and relative flow of the polymer chains during the device deformation. The performance of flexible OSCs has been evaluated for varied stretch levels, from 7% [22] to 22% [24,125], which suggests that significant changes in the length of the polymer chains are somehow possible in real devices [41]. These changes can be even more pronounced with the use of elastomer-based composites [16].

## 4. Conclusions

The effects of mechanical deformations on the structural, optical, and electronic properties of polyaniline (PANI), polyethylene (PT), poly (*p*-phenylene vinylene) (PPV), and polypyrrole (PPy) were evaluated via DFT calculations. 

The results suggest a strong influence of polymer chain stretching on the optoelectronic properties of the systems. In general, the stretching causes a decrease in the energy of the frontier molecular orbitals, with different ratios for the LUMO and HOMO, resulting in an increase in the electronic gaps. Significant hypsochromic effects are induced on the optical absorption spectra, which depend on the evolution of specific chemical bonds. Significant changes were also noticed on the exciton binding and reorganization energies, with small influences on the local reactivity of the systems.

These effects can lead to significant changes in the performance of the materials in devices where polymer elongation can take place, mainly those based on high-molecular-weight compounds and/or all polymer-based devices.

## Figures and Tables

**Table 1:** Structural data of the systems before the deformations and at the point of imminent rupture.

Compound	*d_0_* (Å)	*d_u_* (Å)	Δ*d_max_* (%)
PANI	77.447	92.162	18.0
PT	57.192	69.774	21.0
PPV	94.046	110.974	17.0
PPy	52.588	62.054	17.0

**Table 2:** Relative changes in the total energies (Δ*E_total_*) and estimated values for the forces (*F*) and average elastic constants (*k*).

System	Δ*d_n_* (%)	|Δ*E_total_*| (Joule × 10^−19^)	Force (nN)	Elastic Constant (nN/nm)
PANI	0%	---	---	~5.82
	6%	0.818	3.32	
	12%	2.995	6.01	
	17%	6.325	7.86	
PT	0%	---	---	*k*_1_ = ~10.41 * k_2_ = ~5.66 * k_med_ = ~8.04
	7%	0.582	3.59	
	14%	2.700	4.53	
	20%	5.801	8.37	
PPV	0%	---	---	~5.49
	5%	0.146	1.44	
	11%	1.937	4.79	
	16%	5.379	6.84	
PPy	0%	---	---	*k*_1_ = ~13.87 * k_2_ = ~7.46 * k_med_ = ~10.67
	5%	0.462	3.64	
	11%	2.219	4.31	
	16%	4.808	8.76	

* See Supplementary Materials.

## Data Availability

Not applicable.

## Supplementary Materials

The following supporting information can be downloaded at: https://www.mdpi.com/article/10.3390/polym14071354/s1, Table S1. Bond lengths observed in relaxed polymer structures (at d_0_); Table S2. Summary of the optoelectronic properties of relaxed polymeric systems (at d_0_); Table S3. Effect of XC functional and basis set on the description of optoelectronic properties of the polymers; Table S4. Optical absorption data associated with the main transitions of PANI at distinct levels of deformation (TD-DFT/B3LYP/6-31G approach); Table S5. Optical absorption data associated with the main transitions of PT at distinct levels of deformation (TD-DFT/B3LYP/6-31G approach); Table S6. Optical absorption data associated with the main transitions of PPV at distinct levels of deformation (TD-DFT/B3LYP/6-31G approach); Table S7. Optical absorption data associated with the main transitions of PPy at distinct levels of deformation (TD-DFT/B3LYP/6-31G approach); Figure S1. Deformation of amorphous structures: from amorphous to free optimization and from amorphous to stretched structures; Figure S2. (a) PANI, (b) PT, (c) PPV, and (d) PPy structures at the initial stages of deformation until stabilization; Figure S3. Total energy of the structures: amorphous, full optimization and disentanglement process. (a) PANI, (b) PT, (c) PPV, and (d) PPy; Figure S4. Force imposed to the systems during the disentanglement process: (a) PANI, (b) PT, (c) PPV, and (d) PPy; Figure S5. FMOs of the systems, (a) PANI, (b) PT, (c) PPV, and (d) PPy; Figure S6. Total energy variation (a) PANI, (d) PT; Strength and elastic constant (b) PANI and (e) PT; Figure S7. Total energy variation (a) PPV, (d) PPy, Strength and elastic constant (b) PPV and (e) PPy; Figure S8. DOS for (a) PANI, (b) PT, (c) PPV, and (d) PPy at all levels of stretching; Figure S9. Peak absorption wavelength at all stretch levels; Figure S10. Electronic gap estimated for PT at distinct stretching levels in comparison with Th and Th-2H units; Figure S11. Alignments between the FMO levels of PPV and PT in relation to C_60_ and PCBM. Reference [126,127,128,129,130,131,132,133,134,135,136] are cited in the supplementary materials.

## References

[1] Chen J., Peng Q., Peng X., Han L., Wang X., Wang J., Zeng H. (2020). Recent Advances in Mechano-Responsive Hydrogels for Biomedical Applications. ACS Appl. Polym. Mater..

[2] Lee S., Kim K.Y., Jung S.H., Lee J.H., Yamada M., Sethy R., Kawai T., Jung J.H. (2019). Finely Controlled Circularly Polarized Luminescence of a Mechano-Responsive Supramolecular Polymer. Angew. Chem..

[3] Uǧur G., Chang J., Xiang S., Lin L., Lu J. (2012). A Near-Infrared Mechano Responsive Polymer System. Adv. Mater..

[4] Schäfer C.G., Gallei M., Zahn J.T., Engelhardt J., Hellmann G.P., Rehahn M. (2013). Reversible Light-, Thermo-, and Mechano-Responsive Elastomeric Polymer Opal Films. Chem. Mater..

[5] Willis-Fox N., Rognin E., Aljohani T., Daly R. (2018). Polymer Mechanochemistry: Manufacturing Is Now a Force to Be Reckoned with. Chem.

[6] Takacs L. (2013). The historical development of mechanochemistry. Chem. Soc. Rev..

[7] Crawford D.E., Casaban J. (2016). Recent Developments in Mechanochemical Materials Synthesis by Extrusion. Adv. Mater..

[8] de Oliveira P.F.M., Torresi R.M., Emmerling F., Camargo P.H.C. (2020). Challenges and opportunities in the bottom-up mechanochemical synthesis of noble metal nanoparticles. J. Mater. Chem. A.

[9] Szczęśniak B., Borysiuk S., Choma J., Jaroniec M. (2020). Mechanochemical synthesis of highly porous materials. Mater. Horiz..

[10] Boldyreva E. (2013). Mechanochemistry of inorganic and organic systems: What is similar, what is different?. Chem. Soc. Rev..

[11] Ma R., Feng J., Yin D., Sun H.-B. (2017). Highly efficient and mechanically robust stretchable polymer solar cells with random buckling. Org. Electron..

[12] Ostroverkhova O. (2016). Organic Optoelectronic Materials: Mechanisms and Applications. Chem. Rev..

[13] Lee S.-M., Kwon J.H., Kwon S., Choi K.C. (2017). A Review of Flexible OLEDs Toward Highly Durable Unusual Displays. IEEE Trans. Electron Devices.

[14] Lee H.B., Jin W.-Y., Ovhal M.M., Kumar N., Kang J.-W. (2018). Flexible transparent conducting electrodes based on metal meshes for organic optoelectronic device applications: A review. J. Mater. Chem. C.

[15] Verboven I., Deferme W. (2020). Printing of flexible light emitting devices: A review on different technologies and devices, printing technologies and state-of-the-art applications and future prospects. Prog. Mater. Sci..

[16] Boratto M.H., Nozella N.L., Ramos R.A., Da Silva R.A., Graeff C.F.O. (2020). Flexible conductive blend of natural rubber latex with PEDOT: PSS. APL Mater..

[17] Shen J., Fujita K., Matsumoto T., Hongo C., Misaki M., Ishida K., Mori A., Nishino T. (2017). Mechanical, Thermal, and Electrical Properties of Flexible Polythiophene with Disiloxane Side Chains. Macromol. Chem. Phys..

[18] Chen Q., Wang X., Chen F., Zhang N., Ma M. (2019). Extremely strong and tough polythiophene composite for flexible electronics. Chem. Eng. J..

[19] Etxebarria I., Ajuria J., Pacios R. (2015). Solution-processable polymeric solar cells: A review on materials, strategies and cell architectures to overcome 10%. Org. Electron..

[20] Grimsdale A.C., Chan K.L., Martin R.E., Jokisz P.G., Holmes A.B. (2009). Synthesis of Light-Emitting Conjugated Polymers for Applications in Electroluminescent Devices. Chem. Rev..

[21] Zhang Z., Liao M., Lou H., Hu Y., Sun X., Peng H. (2018). Conjugated Polymers for Flexible Energy Harvesting and Storage. Adv. Mater..

[22] Kim T., Kim J.-H., Kang T.E., Lee C., Kang H., Shin M., Wang C., Ma B., Jeong U., Kim T.-S. (2015). Flexible, Highly Efficient All-Polymer Solar Cells. Nat. Commun..

[23] Savagatrup S., Makaram A.S., Burke D.J., Lipomi D.J. (2013). Mechanical Properties of Conjugated Polymers and Polymer-Fullerene Composites as a Function of Molecular Structure. Adv. Funct. Mater..

[24] Kaltenbrunner M., White M., Głowacki E.D., Sekitani T., Someya T., Sariciftci N.S., Bauer S. (2012). Ultrathin and lightweight organic solar cells with high flexibility. Nat. Commun..

[25] Lee Y., Mongare A., Plant A., Ryu D. (2021). Strain–Microstructure–Optoelectronic Inter-Relationship toward Engineering Mechano-Optoelectronic Conjugated Polymer Thin Films. Polymers.

[26] Fukuda K., Yu K., Someya T. (2020). The Future of Flexible Organic Solar Cells. Adv. Energy Mater..

[27] Schwartz B.J. (2003). Conjugated Polymers as Molecular Materials: How Chain Conformation and Film Morphology Influence Energy Transfer and Interchain Interactions. Annu. Rev. Phys. Chem..

[28] Meier H., Stalmach U., Kolshorn H. (1997). Effective conjugation length and UV/vis spectra of oligomers. Acta Polym..

[29] Rissler J. (2004). Effective conjugation length of π-conjugated systems. Chem. Phys. Lett..

[30] He J., Crase J.L., Wadumethrige S.H., Thakur K., Dai L., Zou S., Rathore R., Hartley C.S. (2010). Ortho-Phenylenes: Unusual Conjugated Oligomers with a Surprisingly Long Effective Conjugation Length. J. Am. Chem. Soc..

[31] Wiebeler C., Gopalakrishna Rao A., Gärtner W., Schapiro I. (2019). The Effective Conjugation Length is Responsible for the Red/Green Spectral Tuning in the Cyanobacteriochrome Slr1393g3. Angew. Chem. Int. Ed..

[32] Kwak K., Cho K., Kim S. (2013). Stable Bending Performance of Flexible Organic Light-Emitting Diodes Using IZO Anodes. Sci. Rep..

[33] Ma R., Chou S.-Y., Xie Y., Pei Q. (2019). Morphological/nanostructural control toward intrinsically stretchable organic electronics. Chem. Soc. Rev..

[34] Caruso M.M., Davis D.A., Shen Q., Odom S.A., Sottos N.R., White S.R., Moore J.S. (2009). Mechanically-Induced Chemical Changes in Polymeric Materials. Chem. Rev..

[35] Rubner M.F. (1986). Novel optical properties of polyurethane-diacetylene segmented copolymers. Macromolecules.

[36] Chen J.-Y., Hsieh H.-C., Chiu Y.-C., Lee W.-Y., Hung C.-C., Chueh C.-C., Chen W.-C. (2019). Electrospinning-induced elastomeric properties of conjugated polymers for extremely stretchable nanofibers and rubbery optoelectronics. J. Mater. Chem. C.

[37] Chow P.C.Y., Someya T. (2019). Organic Photodetectors for Next-Generation Wearable Electronics. Adv. Mater..

[38] Sagara Y., Traeger H., Li J., Okado Y., Schrettl S., Tamaoki N., Weder C. (2021). Mechanically Responsive Luminescent Polymers Based on Supramolecular Cyclophane Mechanophores. J. Am. Chem. Soc..

[39] Onorato J., Pakhnyuk V., Luscombe C.K. (2016). Structure and design of polymers for durable, stretchable organic electronics. Polym. J..

[40] Wang M., Baek P., Akbarinejad A., Barker D., Travas-Sejdic J. (2019). Conjugated polymers and composites for stretchable organic electronics. J. Mater. Chem. C.

[41] Roldao J.C., Batagin-Neto A., Lavarda F.C., Sato F. (2018). Effects of Mechanical Stretching on the Properties of Conjugated Polymers: Case Study for MEH-PPV and P3HT Oligomers. J. Polym. Sci. Part B Polym. Phys..

[42] Brédas J.L., Street G.B., Thémans B., André J.M. (1985). Organic polymers based on aromatic rings (polyparaphenylene, polypyrrole, polythiophene): Evolution of the electronic properties as a function of the torsion angle between adjacent rings. J. Chem. Phys..

[43] Lee I., Rolston N., Brunner P.-L., Dauskardt R.H. (2019). Hole-Transport Layer Molecular Weight and Doping Effects on Perovskite Solar Cell Efficiency and Mechanical Behavior. ACS Appl. Mater. Interfaces.

[44] Kim W., Ma B.S., Kim Y.H., Kim T.-S. (2021). Mechanical properties of organic semiconductors for flexible electronics. Organic Flexible Electronics.

[45] Rasmussen S.C. (2020). Conjugated and Conducting Organic Polymers: The First 150 Years. ChemPlusChem.

[46] Bhadra S., Singha N.K., Khastgir D. (2007). Electrochemical synthesis of polyaniline and its comparison with chemically synthesized polyaniline. J. Appl. Polym. Sci..

[47] Mandú L.O., Batagin-Neto A. (2018). Chemical sensors based on N-substituted polyaniline derivatives: Reactivity and adsorption studies via electronic structure calculations. J. Mol. Model..

[48] Xing J., Liao M., Zhang C., Yin M., Li D., Song Y. (2017). The effect of anions on the electrochemical properties of polyaniline for supercapacitors. Phys. Chem. Chem. Phys..

[49] Hu Z., Zu L., Jiang Y., Lian H., Liu Y., Li Z., Chen F., Wang X., Cui X. (2015). High Specific Capacitance of Polyaniline/Mesoporous Manganese Dioxide Composite Using KI-H2SO4 Electrolyte. Polymers.

[50] Wang H., Lin J., Shen Z.X. (2016). Polyaniline (PANi) based electrode materials for energy storage and conversion. J. Sci. Adv. Mater. Devices.

[51] Kaloni T.P., Giesbrecht P.K., Schreckenbach G., Freund M.S. (2017). Polythiophene: From Fundamental Perspectives to Applications. Chem. Mater..

[52] Kanal I.Y., Owens S.G., Bechtel J.S., Hutchison G. (2013). Efficient Computational Screening of Organic Polymer Photovoltaics. J. Phys. Chem. Lett..

[53] Huong V.T.T., Nguyen H.T., Tai T.B., Nguyen M.T. (2013). π-Conjugated Molecules Containing Naphtho[2,3-b]thiophene and Their Derivatives: Theoretical Design for Organic Semiconductors. J. Phys. Chem. C.

[54] Nimith K., Satyanarayan M., Umesh G. (2018). Enhancement in fluorescence quantum yield of MEH-PPV:BT blends for polymer light emitting diode applications. Opt. Mater..

[55] Farjamtalab I., Sabbaghi-Nadooshan R. (2016). Current density of anodes, recombination rate and luminance in MEH-PPV, MDMO-PPV, and P3HT polymers in polymer light-emitting diodes. Polym. Sci. Ser. A.

[56] Cernini R., Li X.-C., Spencer G., Holmes A., Moratti S., Friend R. (1997). Electrochemical and optical studies of PPV derivatives and poly(aromatic oxadiazoles). Synth. Met..

[57] Yussuf A., Al-Saleh M., Al-Enezi S., Abraham G. (2018). Synthesis and Characterization of Conductive Polypyrrole: The Influence of the Oxidants and Monomer on the Electrical, Thermal, and Morphological Properties. Int. J. Polym. Sci..

[58] Coleone A.P., Lascane L.G., Batagin-Neto A. (2019). Polypyrrole derivatives for optoelectronic applications: A DFT study on the influence of side groups. Phys. Chem. Chem. Phys..

[59] Bibi S., Ullah H., Ahmad S.M., Ali Shah A.-U., Bilal S., Tahir A.A., Ayub K. (2015). Molecular and Electronic Structure Elucidation of Polypyrrole Gas Sensors. J. Phys. Chem. C.

[60] Ghoorchian A., Alizadeh N. (2018). Chemiresistor gas sensor based on sulfonated dye-doped modified conducting polypyrrole film for high sensitive detection of 2,4,6-trinitrotoluene in air. Sens. Actuators B Chem..

[61] Gierschner J., Cornil J., Egelhaaf H.-J. (2007). Optical Bandgaps of π-Conjugated Organic Materials at the Polymer Limit: Experiment and Theory. Adv. Mater..

[62] Bronze-Uhle E.S., Batagin-Neto A., Lavarda F.C., Graeff C. (2011). Ionizing radiation induced degradation of poly (2-methoxy-5-(2’-ethyl-hexyloxy) -1,4-phenylene vinylene) in solution. J. Appl. Phys..

[63] Batagin-Neto A., Oliveira E.F., Graeff C., Lavarda F.C. (2013). Modelling polymers with side chains: MEH-PPV and P3HT. Mol. Simul..

[64] de Oliveira E.F., Camilo A., da Silva-Filho L.C., Lavarda F.C. (2013). Effect of chemical modifications on the electronic structure of poly(3-hexylthiophene). J. Polym. Sci. Part B Polym. Phys..

[65] Oliveira E.F., Lavarda F.C. (2016). Reorganization energy for hole and electron transfer of poly(3-hexylthiophene) derivatives. Polymer.

[66] Oliveira G.P., Barboza B.H., Batagin-Neto A. (2021). Polyaniline-based gas sensors: DFT study on the effect of side groups. Comput. Theor. Chem..

[67] Coleone A.P., Barboza B.H., Batagin-Neto A. (2021). Polypyrrole derivatives for detection of toxic gases: A theoretical study. Polym. Adv. Technol..

[68] Schaftenaar G., Noordik J.H. (2000). Molden: A pre- and post-processing program for molecular and electronic structures. J. Comput. Aided Mol. Des..

[69] Becke A.D. (1993). Density-functional thermochemistry. III. The role of exact exchange. J. Chem. Phys..

[70] Lee C., Yang W., Parr R.G. (1988). Development of the Colle-Salvetti correlation-energy formula into a functional of the electron density. Phys. Rev. B.

[71] Vosko S.H., Wilk L., Nusair M. (1980). Accurate spin-dependent electron liquid correlation energies for local spin density calculations: A critical analysis. Can. J. Phys..

[72] Stephens P.J., Devlin F.J., Chabalowski C.F., Frisch M.J. (1994). Ab Initio Calculation of Vibrational Absorption and Circular Dichroism Spectra Using Density Functional Force Fields. J. Phys. Chem..

[73] McCormick T.M., Bridges C.R., Carrera E.I., DiCarmine P.M., Gibson G.L., Hollinger J., Kozycz L.M., Seferos D.S. (2013). Conjugated Polymers: Evaluating DFT Methods for More Accurate Orbital Energy Modeling. Macromolecules.

[74] Frisch M.J., Trucks G.W., Schlegel H.B., Scuseria G.E., Robb M.A., Cheeseman J.R., Scalmani G., Barone V., Petersson G.A., Nakatsuji H. (2016). Gaussian 16 Revision A.03.

[75] Beyer M.K. (2000). The mechanical strength of a covalent bond calculated by density functional theory. J. Chem. Phys..

[76] Alves G.G., Oliveira E.F., Batagin-Neto A., Lavarda F.C. (2018). Molecular modeling of low bandgap diblock co-oligomers with π-bridges for applications in photovoltaics. Comput. Mater. Sci..

[77] Zhu L., Yi Y., Chen L., Shuai Z. (2008). Exciton binding energy of electronic polymers: A first principles study. J. Theor. Comput. Chem..

[78] Grasser T., Meller G., Li L., Organic Electronics (2010). Advances in Polymer Science.

[79] Hutchison G.R., Ratner M.A., Marks T.J. (2005). Hopping Transport in Conductive Heterocyclic Oligomers: Reorganization Energies and Substituent Effects. J. Am. Chem. Soc..

[80] Yang W., Mortier W.J. (1986). The use of global and local molecular parameters for the analysis of the gas-phase basicity of amines. J. Am. Chem. Soc..

[81] Geerlings P., De Proft A.F., Langenaeker W. (2003). Conceptual Density Functional Theory. Chem. Rev..

[82] Maia R.A., Ventorim G., Batagin-Neto A. (2019). Reactivity of lignin subunits: The influence of dehydrogenation and formation of dimeric structures. J. Mol. Model..

[83] Alves G.G., Lavarda F.C., Graeff C.F., Batagin-Neto A. (2020). Reactivity of eumelanin building blocks: A DFT study of monomers and dimers. J. Mol. Graph. Model..

[84] Hirshfeld F.L. (1977). Bonded-atom fragments for describing molecular charge densities. Theor. Chim. Acta.

[85] De Proft F., Van Alsenoy C., Peeters A., Langenaeker W., Geerlings P. (2002). Atomic charges, dipole moments, and Fukui functions using the Hirshfeld partitioning of the electron density. J. Comput. Chem..

[86] Roy R.K., Pal S., Hirao K. (1999). On non-negativity of Fukui function indices. J. Chem. Phys..

[87] Chirlian L.E., Francl M. (1987). Atomic charges derived from electrostatic potentials: A detailed study. J. Comput. Chem..

[88] Herráez A. (2007). How to Use Jmol to Study and Present Molecular Structures.

[89] Allouche A.-R. (2010). Gabedit-A graphical user interface for computational chemistry softwares. J. Comput. Chem..

[90] Wang Z., Su K., Fan H., Hu L., Wang X., Li Y., Wen Z. (2007). Mechanical and electronic properties of C60 under structure distortion studied with density functional theory. Comput. Mater. Sci..

[91] Salaneck W.R., Clark D.T., Samuelsen E.J. (2019). Science and Applications of Conducting Polymers: Papers from the Sixth European Industrial Workshop.

[92] Chen X., Liang Q.H., Jiang J., Wong C.K.Y., Leung S.Y., Ye H., Yang D.G., Ren T.L. (2016). Functionalization-induced changes in the structural and physical properties of amorphous polyaniline: A first-principles and molecular dynamics study. Sci. Rep..

[93] Salzner U., Lagowski J., Pickup P., Poirier R. (1998). Comparison of geometries and electronic structures of polyacetylene, polyborole, polycyclopentadiene, polypyrrole, polyfuran, polysilole, polyphosphole, polythiophene, polyselenophene and polytellurophene. Synth. Met..

[94] Fu Y., Shen W., Li M. (2008). Theoretical analysis on the electronic structures and properties of PPV fused with electron-withdrawing unit: Monomer, oligomer and polymer. Polymer.

[95] Cumper C.W.N. (1958). The structures of some heterocyclic molecules. Trans. Faraday Soc..

[96] Roöhrig U.F., Frank I. (2001). First-principles molecular dynamics study of a polymer under tensile stress. J. Chem. Phys..

[97] Garnier L., Gauthier-Manuel B., Van Der Vegte E.W., Snijders J., Hadziioannou G. (2000). Covalent bond force profile and cleavage in a single polymer chain. J. Chem. Phys..

[98] Grandbois M., Beyer M., Rief M., Clausen-Schaumann H., Gaub H.E. (1999). How Strong Is a Covalent Bond?. Science.

[99] Smalø H.S., Rybkin V.V., Klopper W., Helgaker T., Uggerud E. (2014). Mechanochemistry: The Effect of Dynamics. J. Phys. Chem. A.

[100] Ruini A., Caldas M.J., Bussi G., Molinari E. (2002). Solid State Effects on Exciton States and Optical Properties of PPV. Phys. Rev. Lett..

[101] Brédas J.-L., Beljonne D., Coropceanu V., Cornil J. (2004). Charge-Transfer and Energy-Transfer Processes in π-Conjugated Oligomers and Polymers: A Molecular Picture. Chem. Rev..

[102] Nayak P.K. (2013). Exciton binding energy in small organic conjugated molecule. Synth. Met..

[103] Melin J., Aparicio F., Subramanian V., Galván M., Chattaraj P.K. (2004). Is the Fukui Function a Right Descriptor of Hard−Hard Interactions?. J. Phys. Chem. A.

[104] Duan L., Uddin A. (2020). Progress in Stability of Organic Solar Cells. Adv. Sci..

[105] Tyler D.R. (2004). Mechanistic Aspects of the Effects of Stress on the Rates of Photochemical Degradation Reactions in Polymers. J. Macromol. Sci. Part C Polym. Rev..

[106] Li Z., Ye B., Hu X., Ma X., Zhang X., Deng Y. (2009). Facile electropolymerized-PANI as counter electrode for low cost dye-sensitized solar cell. Electrochem. Commun..

[107] Malhotra B., Dhand C., Lakshminarayanan R., Dwivedi N., Mishra S., Solanki P., Venkatesh M., Beuerman R.W., Ramakrishna S., Mayandi V. (2015). Polyaniline-based biosensors. Nanobiosensors Dis. Diagn..

[108] Jayasundara W.S.R., Schreckenbach G. (2020). Theoretical Study of p- and n-Doping of Polythiophene- and Polypyrrole-Based Conjugated Polymers. J. Phys. Chem. C.

[109] Kaloni T.P., Schreckenbach G., Freund M.S. (2015). Structural and Electronic Properties of Pristine and Doped Polythiophene: Periodic versus Molecular Calculations. J. Phys. Chem. C.

[110] Sezen M., Plank H., Fisslthaler E., Chernev B., Zankel A., Tchernychova E., Blümel A., List E.J.W., Grogger W., Pölt P. (2011). An investigation on focused electron/ion beam induced degradation mechanisms of conjugated polymers. Phys. Chem. Chem. Phys..

[111] Chambon S., Rivaton A., Gardette J.-L., Firon M. (2007). Photo- and thermal degradation of MDMO-PPV:PCBM blends. Sol. Energy Mater. Sol. Cells.

[112] Chambon S., Rivaton A., Gardette J.-L., Firon M. (2008). Durability of MDMO-PPV and MDMO-PPV: PCBM blends under illumination in the absence of oxygen. Sol. Energy Mater. Sol. Cells.

[113] Le T.-H., Kim Y., Yoon H. (2017). Electrical and Electrochemical Properties of Conducting Polymers. Polymers.

[114] Ohtsuka T. (2012). Corrosion Protection of Steels by Conducting Polymer Coating. Int. J. Corros..

[115] Li Y. (2012). Molecular Design of Photovoltaic Materials for Polymer Solar Cells: Toward Suitable Electronic Energy Levels and Broad Absorption. Acc. Chem. Res..

[116] Brédas J.-L., Norton J.E., Cornil J., Coropceanu V. (2009). Molecular Understanding of Organic Solar Cells: The Challenges. Acc. Chem. Res..

[117] Dang M.T., Hirsch L., Wantz G., Wuest J.D. (2013). Controlling the Morphology and Performance of Bulk Heterojunctions in Solar Cells. Lessons Learned from the Benchmark Poly(3-hexylthiophene):[6,6]-Phenyl-C61-butyric Acid Methyl Ester System. Chem. Rev..

[118] Proctor C., Kuik M., Nguyen T.-Q. (2013). Charge carrier recombination in organic solar cells. Prog. Polym. Sci..

[119] Kim J.-H., Noh J., Choi H., Lee J.-Y., Kim T.-S. (2017). Mechanical Properties of Polymer–Fullerene Bulk Heterojunction Films: Role of Nanomorphology of Composite Films. Chem. Mater..

[120] Kim W., Choi J., Kim J.-H., Kim T., Lee C., Lee S., Kim M., Kim B.J., Kim T.-S. (2018). Comparative Study of the Mechanical Properties of All-Polymer and Fullerene–Polymer Solar Cells: The Importance of Polymer Acceptors for High Fracture Resistance. Chem. Mater..

[121] Cachaneski-Lopes J.P., Batagin-Neto A. (2021). Effects of Mechanical Deformation on the Optoelectronic Properties of Fullerenes: A DFT Study. J. Nanostructure Chem..

[122] Barboza B.H., Gomes O.P., Batagin-Neto A. (2021). Polythiophene derivatives as chemical sensors: A DFT study on the influence of side groups. J. Mol. Model..

[123] Lascane L.G., Oliveira E.F., Galvão D.S., Batagin-Neto A. (2020). Polyfuran-based chemical sensors: Identification of promising derivatives via DFT calculations and fully atomistic reactive molecular dynamics. Eur. Polym. J..

[124] Savagatrup S., Printz A.D., O’Connor T.F., Zaretski A.V., Rodriquez D., Sawyer E.J., Rajan K.M., Acosta R.I., Root S.E., Lipomi D.J. (2014). Mechanical degradation and stability of organic solar cells: Molecular and microstructural determinants. Energy Environ. Sci..

[125] Lipomi D.J., Tee B.C.-K., Vosgueritchian M., Bao Z. (2011). Stretchable Organic Solar Cells. Adv. Mater..

[126] Geniès E.M., Boyle A., Lapkowski M., Tsintavis C. (1990). Polyaniline: A Historical Survey. Synth. Met..

[127] Ullah H. (2017). Inter-Molecular Interaction in Polypyrrole/TiO2: A DFT Study. J. Alloys Compd..

[128] Greenham N.C., Friend R.H. (1996). Semiconductor Device Physics of Conjugated Polymers. Solid State Physics.

[129] McCall R.P., Ginder J.M., Leng J.M., Ye H.J., Manohar S.K., Masters J.G., Asturias G.E., MacDiarmid A.G., Epstein A.J. (1990). Spectroscopy and Defect States in Polyaniline. Phys. Rev. B.

[130] Arjmandi M., Arjmandi A., Peyravi M., Pirzaman A.K. (2020). First-Principles Study of Adsorption of XCN (X = F, Cl, and Br) on Surfaces of Polyaniline. Russ. J. Phys. Chem..

[131] Hosseini H., Mousavi S.M. (2020). Density Functional Theory Simulation for Cr(VI) Removal from Wastewater Using Bacterial Cellulose/Polyaniline. Int. J. Biol. Macromol..

[132] Ullah H., Shah A.-H.A., Bilal S., Ayub K. (2013). DFT Study of Polyaniline NH_3_, CO_2_, and CO Gas Sensors: Comparison with Recent Experimental Data. J. Phys. Chem. C.

[133] Furukawa Y. (1996). Electronic Absorption and Vibrational Spectroscopies of Conjugated Conducting Polymers. J. Phys. Chem..

[134] Zhang L., Colella N.S., Cherniawski B.P., Mannsfeld S.C.B., Briseno A.L. (2014). Oligothiophene Semiconductors: Synthesis, Characterization, and Applications for Organic Devices. ACS Appl. Mater. Interfaces.

[135] Kaneto K., Yoshino K., Inuishi Y. (1983). Electrical and Optical Properties of Polythiophene Prepared by Electrochemical Polymerization. Solid State Commun..

[136] Bredas J.L., Silbey R., Boudreaux D.S., Chance R.R. (1983). Chain-Length Dependence of Electronic and Electrochemical Properties of Conjugated Systems: Polyacetylene, Polyphenylene, Polythiophene, and Polypyrrole. J. Am. Chem. Soc..

