# Supplementary material for: Effects of Mechanical Deformation on the Opto-Electronic Responses, Reactivity, and Performance of Conjugated Polymers: A DFT Study
Source: Polymers (Basel). 2022 Mar 26;14(7):1354. doi: 10.3390/polym14071354 (PMC9002523; doi:10.3390/polym14071354)
Supplement: Supplementary file 1 [file polymers-14-01354-s001.zip › polymers-1493398-supplementary.pdf]

polymers

MDPI

SUPPLEMENTARY MATERIAL

Effects of mechanical deformation on the optoelectronic responses, reactivity and performance of conjugated polymers: a DFT study

João P. Cachaneski-Lopes [1] and Augusto Batagin-Neto [*,2]

[1] São Paulo State University (UNESP), School of Sciences, POSMAT, Bauru/SP 17033-360, Brazil
[2] São Paulo State University (UNESP), Campus of Itapeva, Itapeva/SP 18409-010, Brazil
* Correspondence: a.batagin@unesp.br

S1. Structural properties

Table S1 presents the average values estimated for the simple (C-C), double (C=C), resonant (C---C) and heteroatomic carbon bond lengths estimated for each polymer along the main chains.

Table S1. Bond lengths observed in relaxed polymer structures (at d_0).

System	Bond length (Å)			
	C-C	C=C	C---C	C-R
PANI	1.400 ± 0.007	---	1.390 ± 5x10^{-4}	1.400 ± 3x10^{-7}
PT	1.420 ± 0.004	1.380 ± 7x10^{-8}	---	1.820 ± 1x10^{-6}
PPV	1.460 ± 9x10^{-8}	1.370 ± 0.020	1.410 ± 8x10^{4}	---
PPy	1.430 ± 0.009	1.390 ± 8x10^{-7}	---	1.390 ± 3x10^{-5}

It is possible to note that the presented data are compatible with those presented in the literature [1–4].

Figure S1 illustrates a snapshot of the systems during a molecular dynamics simulation (NVT ensemble at 300 K during 1000 ps using AMBER force field) as implemented in the Gabedit computational package (labeled as amorphous), fully optimized structures (without restrictions) (labeled as free) and then optimized structures restricting the distances between terminal carbons at different levels of deformation (labeled as disentanglement ones).

In general, it is noted that the structures acquire a very folded conformation during MD (at room temperature), however it converges to the initial structures considered in this work after successive stretching processes. This result reinforces the validity of our results in very stretched systems. When performing the full optimization starting from linear/planarized structures, some structures remain linear/planarized while the others present curved/twisted conformations, as indicated by Figure S2: PPV, PT in PPy have slightly distorted structures (relaxed systems), tending to assume linear/planar conformations after the stretching.

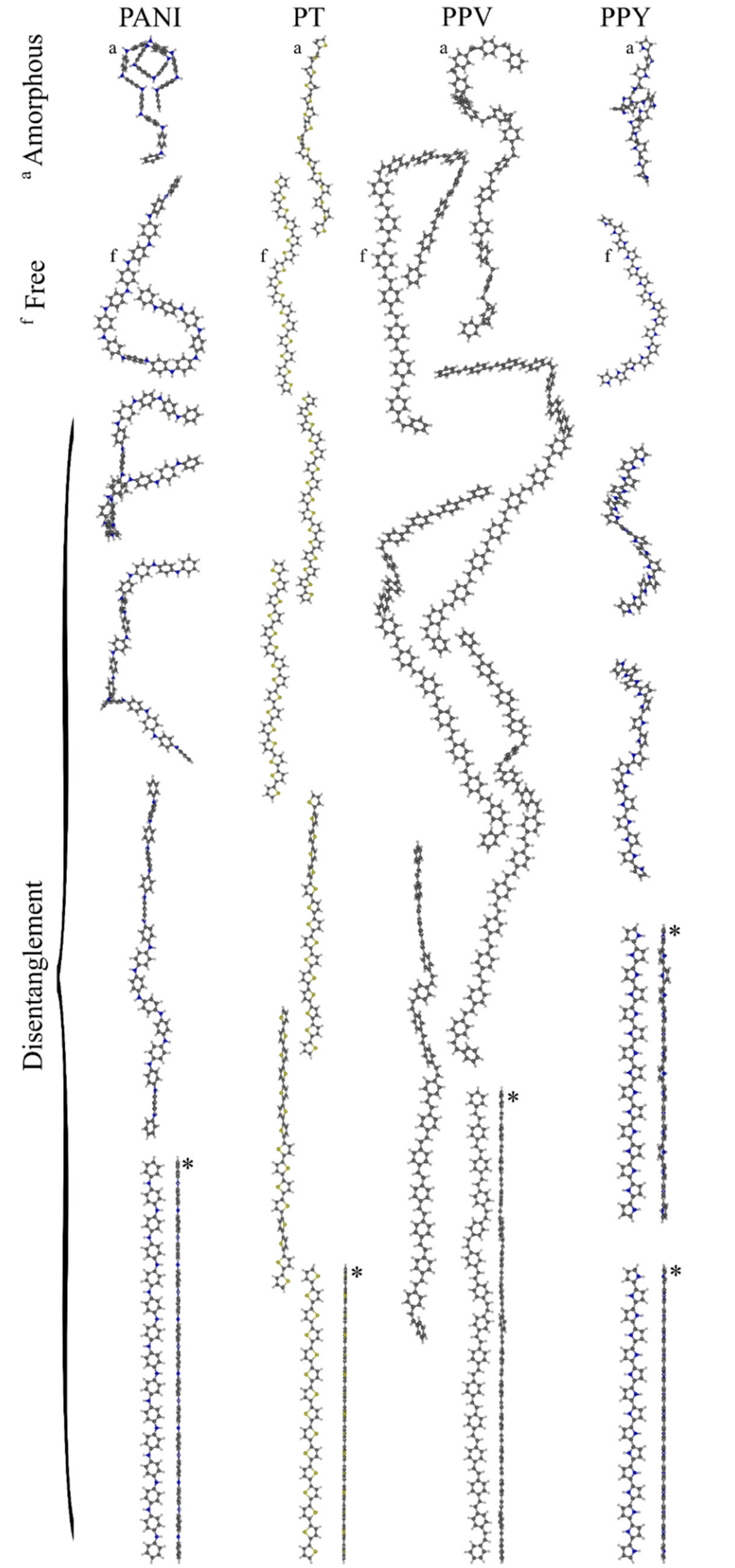

*Frontal view of the structures.

Figure S1. Deformation of amorphous structures: from amorphous to free optimization and from amorphous to stretched structures.

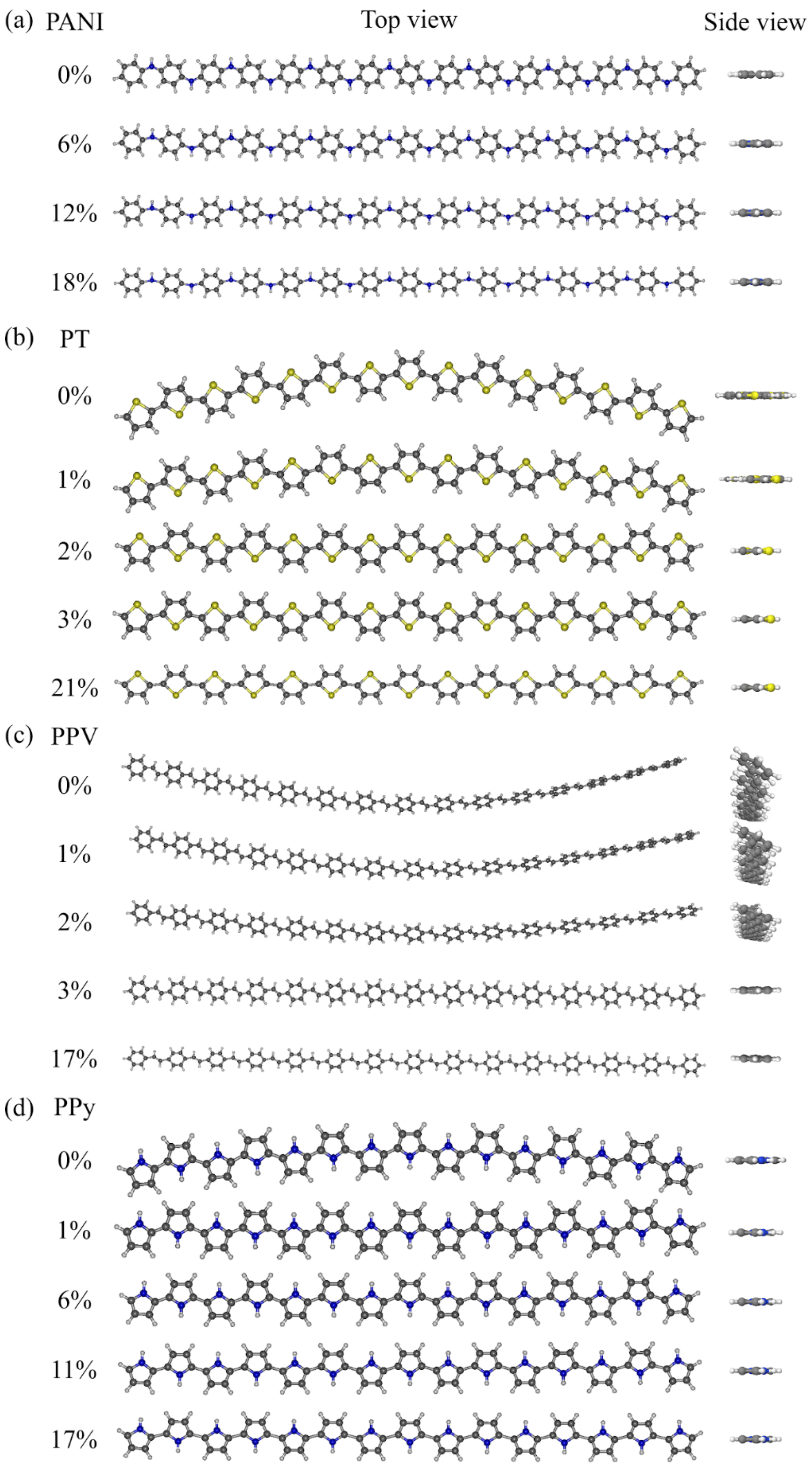

Figure S2. (**a**) PANI, (**b**) PT, (**c**) PPV and (**d**) PPy structures at the initial stages of deformation until stabilization.

S2. Effects of disentanglement on the total energy, force and FMOs

Figure S3 illustrates the total energy of the systems throughout the disentanglement process, from amorphous to fully stretched structures.

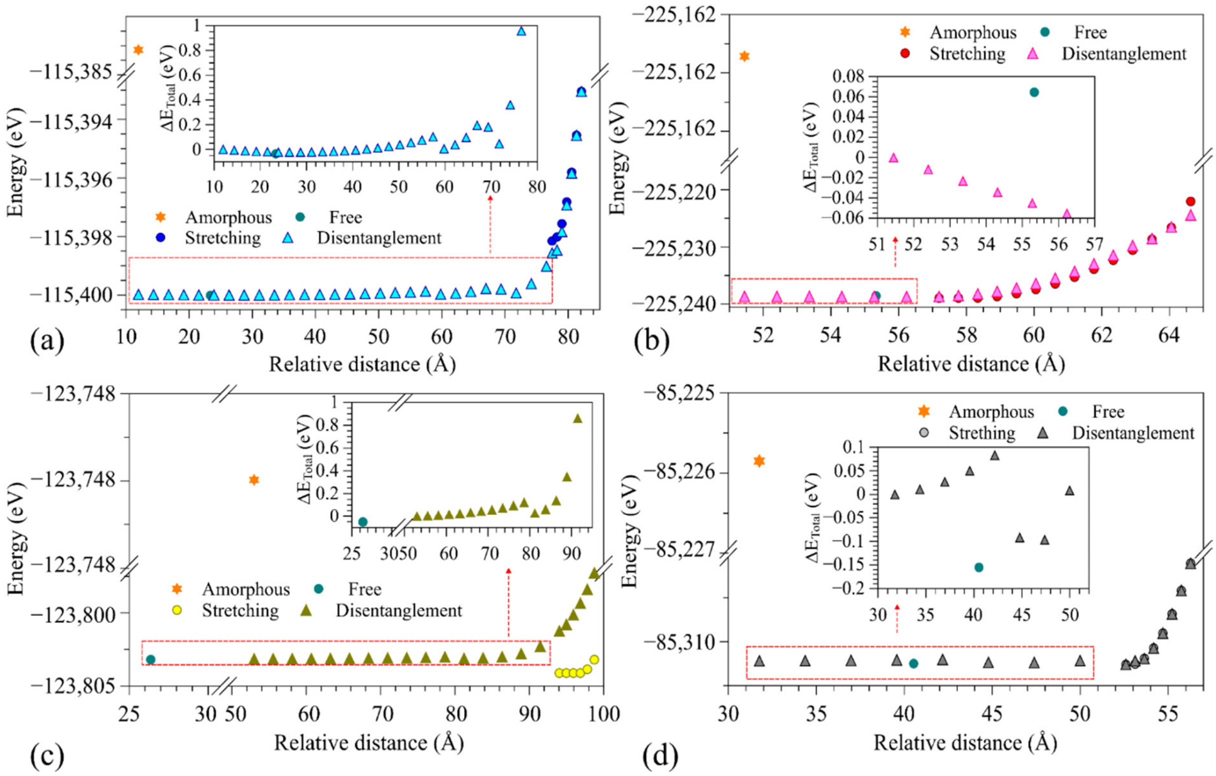

Figure S3. Total energy of the structures: amorphous, full optimization and disentanglement process. (**a**) PANI, (**b**) PT, (**c**) PPV and (**d**) PPy.

Small energy changes are noticed during the disentanglement process (up to ~1 eV), more significant variations are associated with monomers rotation (from synclinal/gauche to antiperiplanar conformations), which are mainly noticed for PANI, PPV and PPy. The differences between the total energy of stretched PPV structures (from disentanglement process *versus* directly stretched ones) can be attributed to the formation of some cis-configurations during the streching process of amorphous structures (see Figure S1).

Figure S4 shows the resulting forces (from the energy variation in relation to the increase of the relative distances) imposed to the systemd during the disentanglement process.

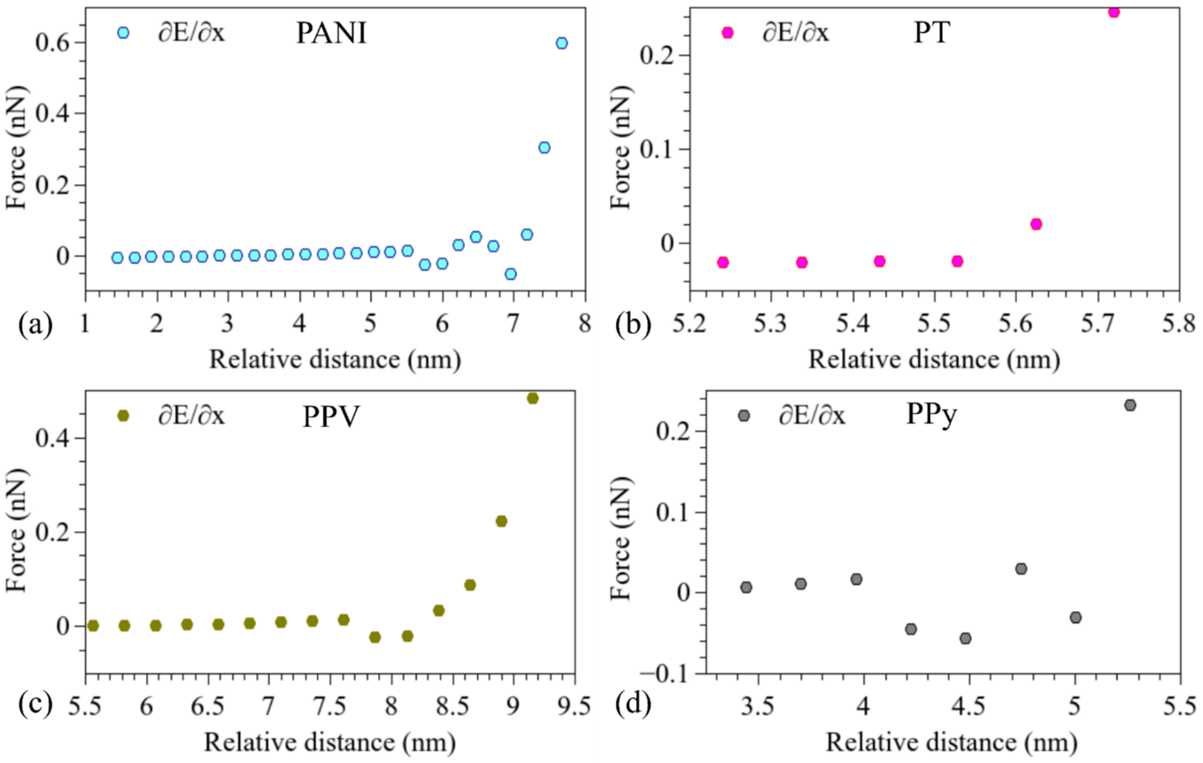

Figure S4. Force imposed to the systems during the disentanglement process: (**a**) PANI, (**b**) PT, (**c**) PPV and (**d**) PPy.

Note that external forces around 0.7 nN are necessary to promote the disentanglement of the systems, the most significant variations are associated with the rotation of monomers, leading to relaxation effects (slight negative forces).

Figure S5 shows the evolution of the frontier molecular orbitals during the disentanglement process (from free to stretched) compared to stretched systems (from initial planar chains) presented in the main text.

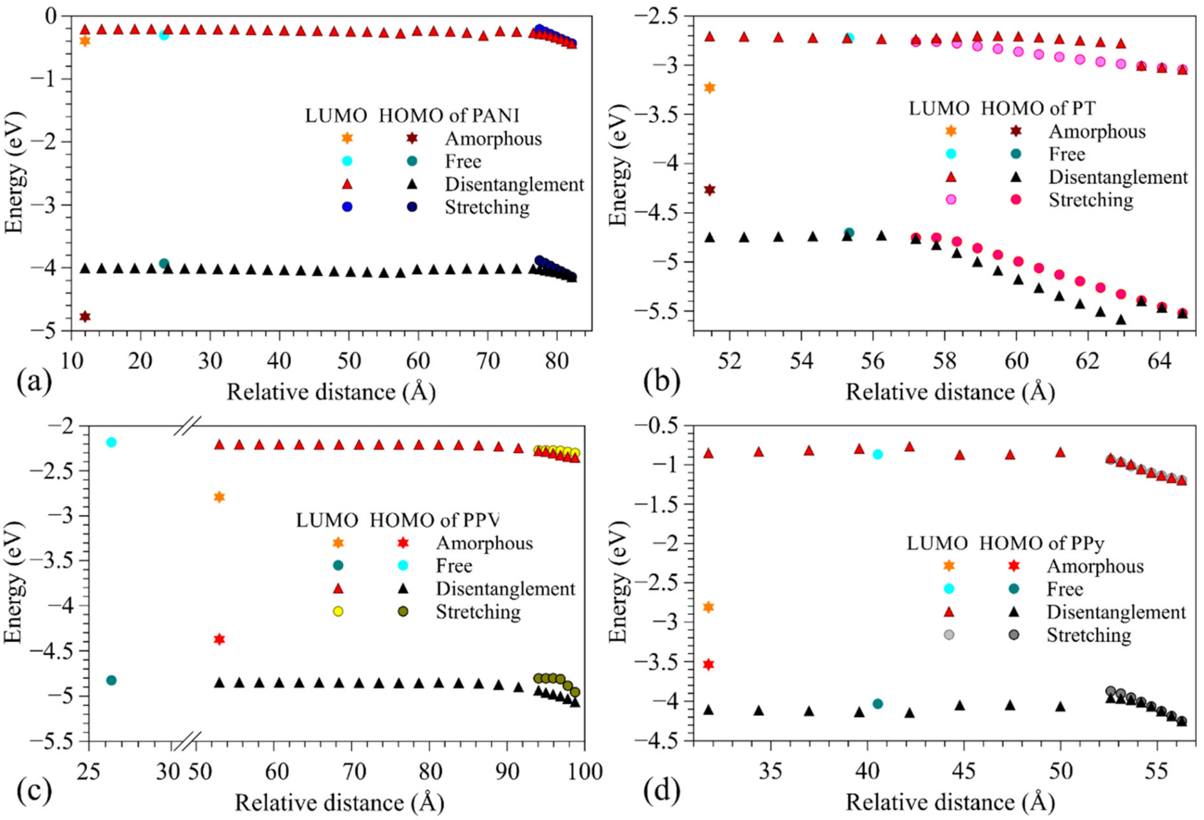

Figure S5. FMOs of the systems, (a) Pani, (b) PT, (c) PPV and (d) PPy.

Amorphous structures present dissimilar bandgaps in relation to the free structures and those produced during the disentanglement process. After the stretching they converge to those obtained from initial planarized structures.

S3. Mechanical properties of stretched systems

Figures S6 and S7 show the changes induced on the total energy of the systems ($\Delta E_{total} = E_{total}^{(n)} - E_{total}^{(0)}$) for the distinct deformation levels ($x = d_n - d_0$). The amplitudes of the forces ($|F|$) required for each level of deformation were estimated by $|F|=d\Delta E_{total}/dx$. The elastic constants (k) were estimated via linear fittings, by considering $|F|=k.x$.

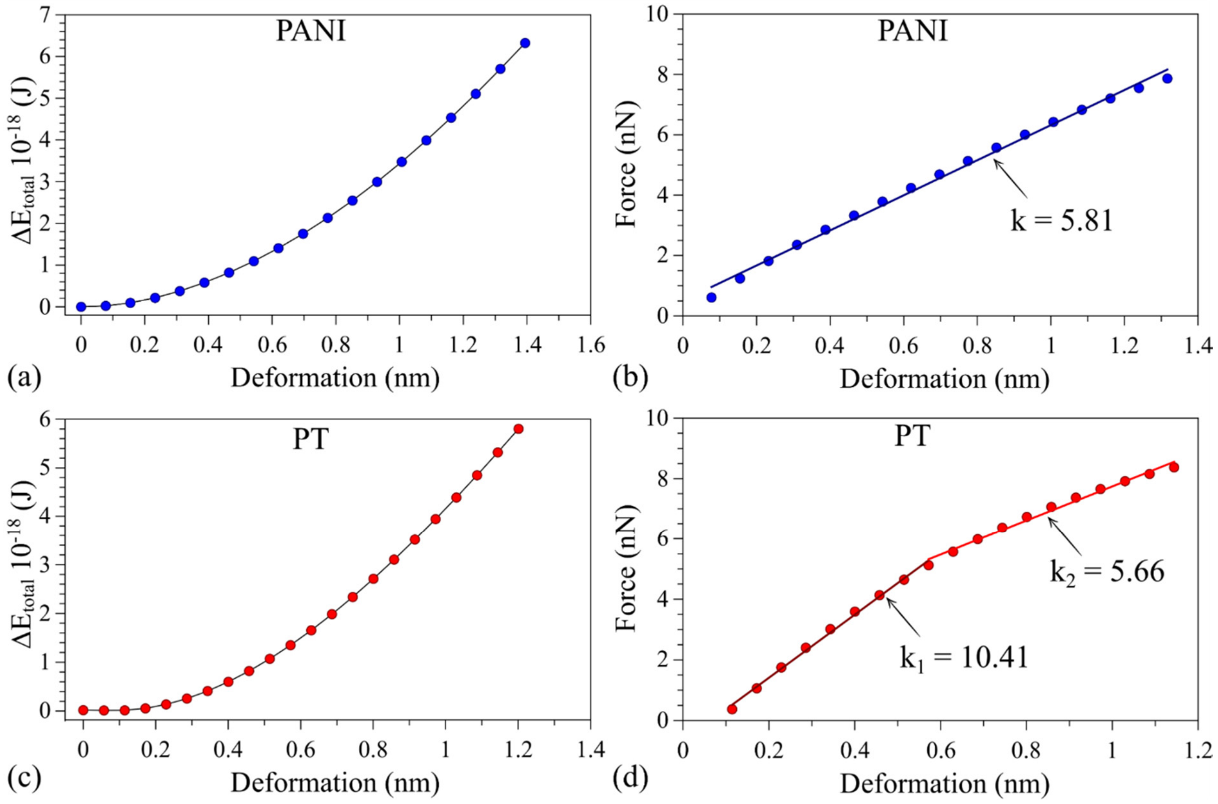

Figure S6. Total energy variation (a) PANI, (d) PT; Strength and elastic constant (b) PANI and (e) PT.

Similar behaviors are noticed for benzene-based polymers (PANI and PPV) and five-membered rings (PT and PPy). For PANI and PPV, it is observed that *F* is well described by a single straight line in relation to the deformation, leading to well defined values of the elastic constants (*k*). A different behavior is noted for PT and PPy, where two different regimes are noticed for *F*, leading to higher average values of *k*.

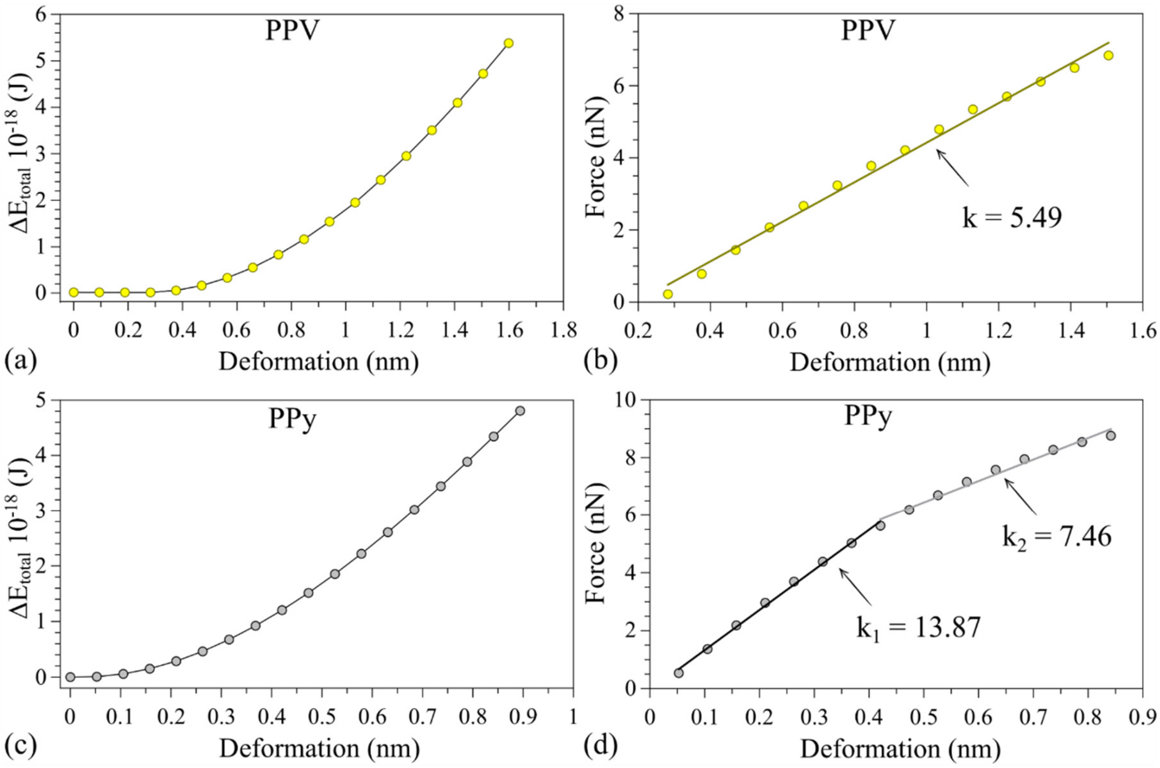

Figure S7. Total energy variation (**a**) PPV, (**d**) PPy, Strength and elastic constant (**b**) PPV and (c) PPy.

S4. Effect of mechanical deformation on the DOS

Figure S8 illustrates the effects of mechanical deformations on the total density of electronic states for all the stretching levels. In general, it is noted, although the most significant variations occur far away from the frontier levels, only with subtle changes around the HOMO and LUMO (mainly in the LUMO).

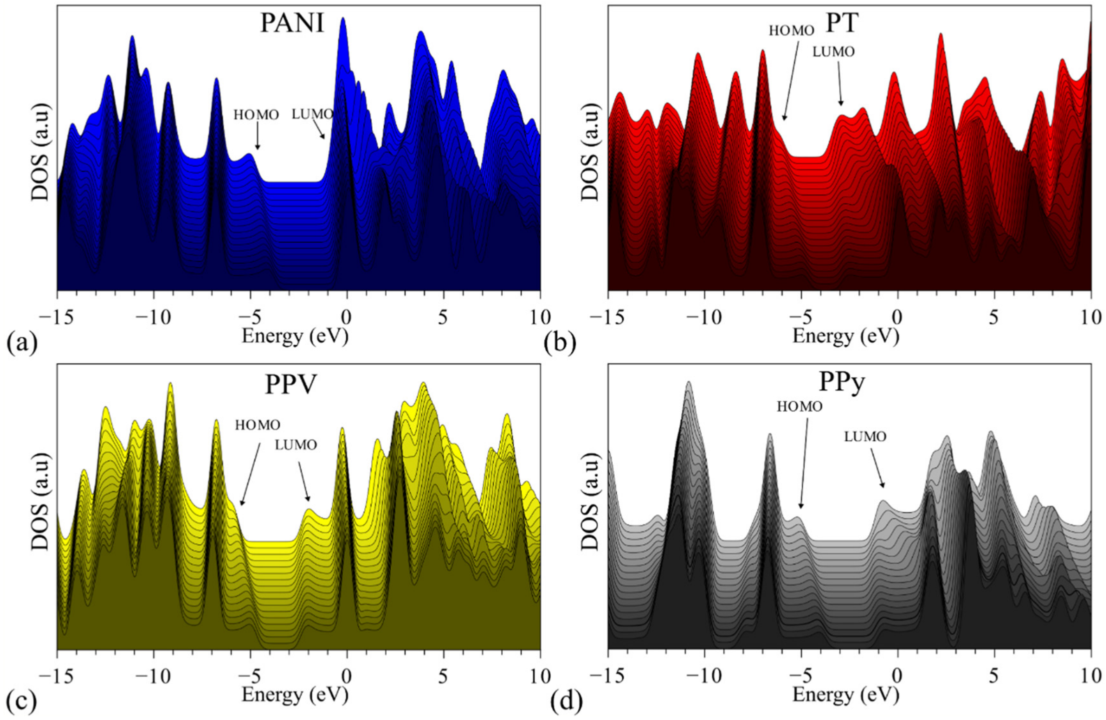

Figure S8. DOS for (**a**) PANI, (**b**) PT, (**c**) PPV and (**d**) PPy at all levels of stretching.

S5. Optoelectronic properties

Table S2 presents the FMO energy levels (E_{HOMO} and E_{LUMO}) and the electronic gaps (E_{gap}), first vertical excitation energies for singlet states (E_{vert}), exciton binding energies (E_X) and internal reorganization energies for holes (λ_h) and electrons (λ_e).

Table S2. Summary of the optoelectronic properties of relaxed polymeric systems (at d_0).

Systems	Energy (eV)						
	E_{HOMO}	E_{LUMO}	E_{gap}	E_{vert}	E_X	λ_h	λ_e
PANI	−3.882	−0.210	3.672	3.232	0.440	0.034	0.029
PT	−4.753	−2.762	1.990	1.701	0.286	0.131	0.116
PPV	−4.804	−2.269	2.534	2.205	0.329	0.057	0.063
PPy	−3.871	−0.934	2.937	2.537	0.397	0.094	0.086

The obtained values are compatible with those reported in the literature for undoped systems [3,5–8].

Table S3. Effect of XC functional and basis set on the description of optoelectronic properties of the polymers.

System	Approach	Deformation	E_{HOMO} (eV)	E_{LUMO} (eV)	E_{gap} (eV)
PANI	Literature [a]	---	from −5.2 to −4.3	from −3.4 to −0.21	from 1.1 to 4.5
	B3LYP/ 6-31G	0%	−3.88	−0.21	3.67
		6%	−4.15 (−0.27)	−0.44 (−0.23)	3.71 (0.04)
	B3LYP/ 6-31G*	0%	−3.86	−0.25	3.61
		6%	−4.13 (−0.27)	−0.48 (−0.23)	3.65 (0.04)
	CAM-B3LYP/ 6-31G	0%	−5.07	0.99	6.06
		6%	−5.36 (−0.29)	0.76 (−0.23)	6.12 (0.06)
	CAM-B3LYP/ 6-31G*	0%	−5.06	0.95	6.01
		6%	−5.35 (−0.29)	0.72 (−0.23)	6.07 (0.06)
PT	Literature [b]	---	from −5.24 to −4.44	from −3.33 to −2.50	from 1.94 to 2.34
	B3LYP/ 6-31G	0%	−4.75	−2.76	1.99
		6%	−5.13 (−0.38)	−2.92 (−0.16)	2.21 (0.22)
	B3LYP/ 6-31G*	0%	−4.63	−2.46	2.17
		6%	−5.01 (−0.38)	−2.62 (−0.16)	2.39 (0.22)
	CAM-B3LYP/6-31G	0%	−6.02	−1.64	4.38
		6%	−6.48 (−0.46)	−1.77 (−0.13)	4.71 (0.33)
	CAM-B3LYP/6-31G*	0%	−5.90	−1.32	4.58
		6%	−6.36 (−0.46)	−1.45 (−0.13)	4.91 (0.33)

Table S3. Continued.

PPV	Literature [c]	---	from −7.01 to −4.47	from −2.85 to −2.76	from 1.71 to 2.55
	B3LYP/ 6-31G	0%	−4.80	−2.27	2.53
		6%	−4.96 (−0.16)	−2.30 (−0.03)	2.66 (0.13)
	B3LYP/ 6-31G*	0%	−4.79	−2.29	2.50
		6%	−4.95 (−0.16)	−2.32 (−0.03)	2.63 (0.13)
	CAM-B3LYP/6-31G	0%	−6.03	−1.10	4.93
		6%	−6.22 (−0.19)	−1.16 (−0.06)	5.06 (0.13)
	CAM-B3LYP/6-31G*	0%	−6.03	−1.10	4.93
		6%	−6.22 (−0.19)	−1.16 (−0.06)	5.06 (0.13)
PPy	Literature [d]	---	from −5.68 to −4.03	from −2.22 to −0.57	from 2.81 to 3.46
	B3LYP/6-31G	0%	−3.87	−0.93	2.94
		6%	−4.13 (−0.26)	−1.14 (−0.21)	2.99 (0.05)
	B3LYP/ 6-31G*	0%	−3.82	−0.84	2.98
		6%	−4.08 (−0.26)	−1.05 (−0.21)	3.03 (0.05)
	CAM-B3LYP/6-31G	0%	−5.09	0.27	5.36
		6%	−5.39 (−0.30)	0.07 (−0.20)	5.46 (0.10)
	CAM-B3LYP/6-31G*	0%	−5.06	0.37	5.43
		6%	−5.36 (−0.30)	0.17 (−0.20)	5.53 (0.10)

[a] From refs: [5,8–12]; [b] From refs: [3,8,13–16]; [c] From refs: [7,8,17]; [d] From refs: [3,6,8,17,18].

As can be seen, the extension of the basis set (from 6-31G to 6-31G*) and the change of the XC functional (B3LYP and CAM-B3LYP) do not lead to significant changes in the results for both, relaxed and stretched systems, as well as on the relative variations (comparison between stretched and relaxed structures presented in parenthesis). In particular, we consider that B3LYP/6-31G data acceptable, given the higher relative computational cost of the 6-31G*-based approach (at least 4x for PANI and PPV, 33x for PT and 2x for PPy for geometry optimization of stretched neutral structures). In particular, regarding the XC functional, it is noted that CAM-B3LYP presents good results in relation to the absolute values of E_{HOMO} for PPV and PPy, however, it leads to significant deviations for E_{gap}, in comparison with B3LYP for all systems in relation to the literature. In addition to the well known ability of B3LYP to describe structural, electronic, and optical properties of medium size conjugated systems [19], the above presented results have motivated the choice of B3LYP/6-31G approach in this study.

Tables S4-S7 show the first excited states of PANI, PT, PPV and PPy, respectively, for distinct levels of deformation.

Table S4. Optical absorption data associated with the main transitions of PANI at distinct levels of deformation (TD-DFT/B3LYP/6-31G approach).

**Deformation (%).**	E_{vert} **(eV)**	λ_{max} **(nm)**	f_{osc}	**Main transitions**	$c_i^2 \times 100$
0%	3.232	384.06	7.721	$H_2 \rightarrow L$	2.518
				$H_1 \rightarrow L_1$	8.113
				$H_1 \rightarrow L_{10}$	1.208
				$H \rightarrow L$	32.500
				$H \rightarrow L_5$	3.488
6%	3.267	379.50	8.307	$H_2 \rightarrow L_2$	2.870
				$H_1 \rightarrow L$	8.505
				$H_1 \rightarrow L_3$	1.164
				$H \rightarrow L$	31.780
				$H \rightarrow L_2$	3.018
12%	3.360	368.98	8.759	$H_3 \rightarrow L_3$	1.314

				$H_2 \rightarrow L_2$	3.434
				$H_1 \rightarrow L_1$	9.105
				$H_1 \rightarrow L_3$	1.132
				$H \rightarrow L$	30.631
18%	3.519	352.35	8.837	$H_3 \rightarrow L_3$	1.984
				$H_2 \rightarrow L_2$	4.465
				$H_1 \rightarrow L_1$	9.902
				$H \rightarrow L$	27.881
				$H \rightarrow L_2$	1.243

Table S5. Optical absorption data associated with the main transitions of PT at distinct levels of deformation (TD-DFT/B3LYP/6-31G approach).

Deformation (%)	E_{vert} (eV)	λ_{max} (nm)	f_{osc}	Main transitions	$c_i^2 \times 100$
0%	1.705	726.84	5.215	$H_1 \rightarrow L_1$	2.928
				$H \rightarrow L$	46.425
7%	1.905	650.69	5.537	$H_1 \rightarrow L_1$	3.538
				$H \rightarrow L$	45.402
14%	2.187	566.85	5.530	$H_1 \rightarrow L_1$	4.720
				$H \rightarrow L$	43.235
20%	2.508	494.33	5.262	$H_2 \rightarrow L_2$	1.398
				$H_2 \rightarrow L_6$	1.357
				$H_1 \rightarrow L_4$	6.147
				$H \rightarrow L_2$	39.999
				$H \rightarrow L$	32.039

Table S6. Optical absorption data associated with the main transitions of PPV at distinct levels of deformation (TD-DFT/B3LYP/6-31G approach).

Deformation (%)	E_{vert} (eV)	λ_{max} (nm)	f_{osc}	Main transitions	$c_i^2 \times 100$
0%	2.205	562.29	9.583	$H_2 \rightarrow L_2$	2.006
				$H_1 \rightarrow L_1$	7.347
				$H \rightarrow L$	38.422
5%	2.32	534.47	10.164	$H_2 \rightarrow L_2$	2.217
				$H_1 \rightarrow L_1$	7.735
				$H \rightarrow L$	37.473
11%	2.665	465.26	10.481	$H_3 \rightarrow L_3$	1.093
				$H_2 \rightarrow L$	1.088
				$H_2 \rightarrow L_2$	3.039
				$H_1 \rightarrow L_1$	8.956
				$H \rightarrow L$	33.955
17%	3.162	392.10	10.422	$H_3 \rightarrow L_3$	1.757
				$H_2 \rightarrow L_6$	1.759
				$H_2 \rightarrow L_2$	4.186
				$H_1 \rightarrow L_1$	9.902
				$H \rightarrow L$	29.021

Table S7. Optical absorption data associated with the main transitions of PPy at distinct levels of deformation (TD-DFT/B3LYP/6-31G approach).

Deformation (%)	E_{vert} (eV)	λ_{max} (nm)	f_{osc}	Main transitions	$c_i^2 \times 100$
0%	2.540	488.15	4.850	$H_1 \rightarrow L_1$	4.967
				$H \rightarrow L$	43.295
5%	2.540	476.86	5.125	$H_1 \rightarrow L_1$	4.958
				$H \rightarrow L$	43.217
11%	2.872	431.74	5.160	$H_2 \rightarrow L_2$	1.247
				$H_1 \rightarrow L_1$	5.859
				$H \rightarrow L$	41.505
17%	3.403	364.36	4.972	$H_2 \rightarrow L$	1.272
				$H_2 \rightarrow L_2$	1.987
				$H_1 \rightarrow L_1$	7.588
				$H \rightarrow L$	37.567

Figure S9. shows the evolution of the main peak positions in relation to deformation levels for the distinct polymers. Note that the shifts follow the increasing order: PANI < PPy < PPV < PT. Linear behaviors are noticed for PT and PPV, with non-linear responses for PANI and PPy.

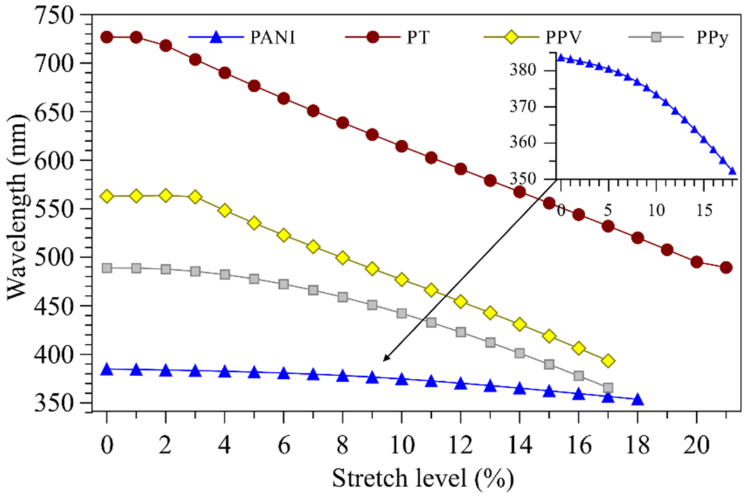

Figure S9. Peak absorption wavelength at all stretch levels.

S6. Effect of mechanical deformation on the electronic gap of PT

Figure S10 illustrates the changes noticed on the electronic gap of PT (at 0%, 19% and 21%) in comparison with the isolated thiophene unit (Th) and non-passivated thiophene ring (Th-2H) (see the structures in the inset). The geometry optimization and single point calculations for the estimation of KS-frontier level energies were conducted using the same theoretical approach employed for PT (15 units) oligomer.

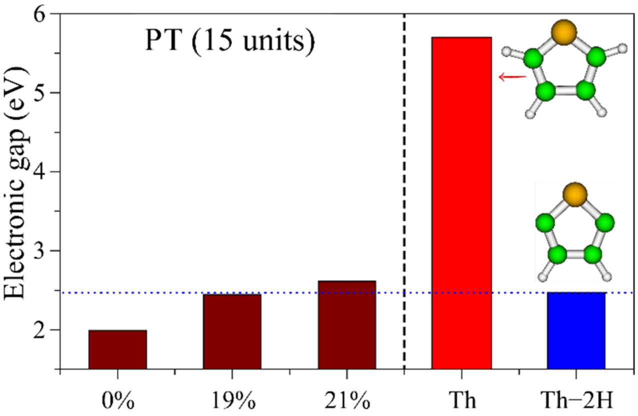

Figure S10. Electronic gap estimated for PT at distinct stretching level in comparison with Th and Th-2H units.

Note that during the stretching process the electronic gap of the oligomer tend to those obtained for non-passivated Th-2H structures. The best match occurs at 19% when a transitional behavior is observed for PT (see Figure 5 in the main text).

S7. Parameters associated with the performance of BHJ-OSCs

Additional calculations were conducted to estimate the electronic properties of typical semiconducting acceptor: fullerene (C_{60}) and [6,6]-phenyl-C_{61} butyric methyl ester (PCBM). The frontier energy levels were estimated using the same approach employed to study the polymeric chains (DFT/B3LYP/6-31G).

Figure S11 shows the changes induced on the relative alignments between the FMOs of the PPV and PT (commonly used in BHJ-OSCs). Details on the evolution of typical BHJ-OSC performance parameters (open circuit voltage, V_{OC}; and differences in the energies of $HOMO_{donor}$-$HOMO_{acceptor}$ and $LUMO_{donor}$-$LUMO_{acceptor}$, ΔE_{HH} and ΔE_{LL}) are presented in the main text (Figs 14 and 15).

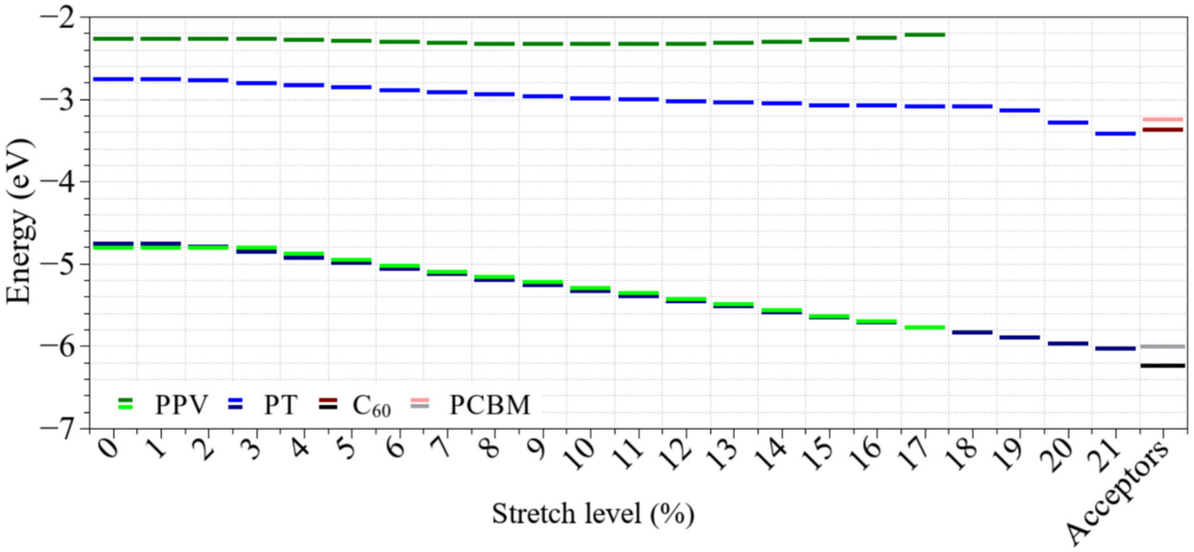

Figure S11. Alignments between the FMO levels of PPV and PT in relation to C_{60} and PCBM.

References

1. Chen, X.P.; Liang, Q.H.; Jiang, J.K.; Wong, C.K.Y.; Leung, S.Y.Y.; Ye, H.Y.; Yang, D.G.; Ren, T.L. Functionalization-Induced Changes in the Structural and Physical Properties of Amorphous Polyaniline: A First-Principles and Molecular Dynamics Study. Sci. Rep. 2016, 6, 20621, doi:10.1038/srep20621.
2. SALANECK Science and Applications of Conducting Polymers, Papers from the Sixth European Industrial Workshop; CRC Press: Place of publication not identified, 2019; ISBN 978-1-00-011224-5.
3. Salzner, U.; Lagowski, J.B.; Pickup, P.G.; Poirier, R.A. Comparison of Geometries and Electronic Structures of Polyacetylene, Polyborole, Polycyclopentadiene, Polypyrrole, Polyfuran, Polysilole, Polyphosphole, Polythiophene, Polyselenophene and Polytellurophene. Synth. Met. 1998, 96, 177–189, doi:10.1016/S0379-6779(98)00084-8.
4. Fu, Y.; Shen, W.; Li, M. Theoretical Analysis on the Electronic Structures and Properties of PPV Fused with Electron-Withdrawing Unit: Monomer, Oligomer and Polymer. Polym. 2008, 49, 2614–2620, doi:10.1016/j.polymer.2008.03.051.
5. Geniès, E.M.; Boyle, A.; Lapkowski, M.; Tsintavis, C. Polyaniline: A Historical Survey. Synth. Met. 1990, 36, 139–182, doi:10.1016/0379-6779(90)90050-U.
6. Ullah, H. Inter-Molecular Interaction in Polypyrrole/TiO2: A DFT Study. J. Alloys Compd. 2017, 692, 140–148, doi:10.1016/j.jallcom.2016.08.169.
7. Cernini, R.; Li, X.-C.; Spencer, G.W.C.; Holmes, A.B.; Moratti, S.C.; Friend, R.H. Electrochemical and Optical Studies of PPV Derivatives and Poly(Aromatic Oxadiazoles). Synth. Met. 1997, 84, 359–360, doi:10.1016/S0379-6779(97)80781-3.
8. Greenham, N.C.; Friend, R.H. Semiconductor Device Physics of Conjugated Polymers. In Solid State Physics; Elsevier, 1996; Vol. 49, pp. 1–149 ISBN 978-0-12-607749-0.
9. McCall, R.P.; Ginder, J.M.; Leng, J.M.; Ye, H.J.; Manohar, S.K.; Masters, J.G.; Asturias, G.E.; MacDiarmid, A.G.; Epstein, A.J. Spectroscopy and Defect States in Polyaniline. Phys. Rev. B 1990, 41, 5202–5213, doi:10.1103/PhysRevB.41.5202.

10. Mehrzad Arjmandi; Arjmandi, A.; Peyravi, M.; Pirzaman, A.K. First-Principles Study of Adsorption of XCN (X = F, Cl, and Br) on Surfaces of Polyaniline. Russ. J. Phys. Chem. 2020, 94, 2148–2154, doi:10.1134/S0036024420100027.
11. Hosseini, H.; Mousavi, S.M. Density Functional Theory Simulation for Cr(VI) Removal from Wastewater Using Bacterial Cellulose/Polyaniline. Int. J. Biol. Macromol. 2020, 165, 883–901, doi:10.1016/j.ijbiomac.2020.09.217.
12. Ullah, H.; Shah, A.-H.A.; Bilal, S.; Ayub, K. DFT Study of Polyaniline NH_3, CO_2, and CO Gas Sensors: Comparison with Recent Experimental Data. J. Phys. Chem. C 2013, 117, 23701–23711, doi:10.1021/jp407132c.
13. Furukawa, Y. Electronic Absorption and Vibrational Spectroscopies of Conjugated Conducting Polymers. J. Phys. Chem. 1996, 100, 15644–15653, doi:10.1021/jp960608n.
14. Barboza, B.H.; Gomes, O.P.; Batagin-Neto, A. Polythiophene Derivatives as Chemical Sensors: A DFT Study on the Influence of Side Groups. J. Mol. Model. 2021, 27, 17, doi:10.1007/s00894-020-04632-w.
15. Zhang, L.; Colella, N.S.; Cherniawski, B.P.; Mannsfeld, S.C.B.; Briseno, A.L. Oligothiophene Semiconductors: Synthesis, Characterization, and Applications for Organic Devices. ACS Appl. Mater. Interfaces 2014, 6, 5327–5343, doi:10.1021/am4060468.
16. Kaneto, K.; Yoshino, K.; Inuishi, Y. Electrical and Optical Properties of Polythiophene Prepared by Electrochemical Polymerization. Solid State Commun. 1983, 46, 389–391, doi:10.1016/0038-1098(83)90454-4.
17. Bredas, J.L.; Silbey, R.; Boudreaux, D.S.; Chance, R.R. Chain-Length Dependence of Electronic and Electrochemical Properties of Conjugated Systems: Polyacetylene, Polyphenylene, Polythiophene, and Polypyrrole. J. Am. Chem. Soc. 1983, 105, 6555–6559, doi:10.1021/ja00360a004.
18. Zotti, G.; Martina, S.; Wegner, G.; Schlüter, A.-D. Well-Defined Pyrrole Oligomers: Electrochemical and UV/Vis Studies. Adv. Mater. 1992, 4, 798–801, doi:10.1002/adma.19920041206.
19. McCormick, T.M.; Bridges, C.R.; Carrera, E.I.; DiCarmine, P.M.; Gibson, G.L.; Hollinger, J.; Kozycz, L.M.; Seferos, D.S. Conjugated Polymers: Evaluating DFT Methods for More Accurate Orbital Energy Modeling. Macromol. 2013, 46, 3879–3886, doi:10.1021/ma4005023.
